# Supplementary material for: Tripartite motif containing 26 prevents steatohepatitis progression by suppressing C/EBPδ signalling activation
Source: Nat Commun. 2023 Oct 11;14:6384. doi: 10.1038/s41467-023-42040-9 (PMC10567751; doi:10.1038/s41467-023-42040-9)
Supplement: Supplementary file 1 — Supplementary information [file 41467_2023_42040_MOESM1_ESM.pdf]

Supplementary information for:

Tripartite motif containing 26 prevents steatohepatitis progression by suppressing C/EBPδ signaling activation

Xu et al.

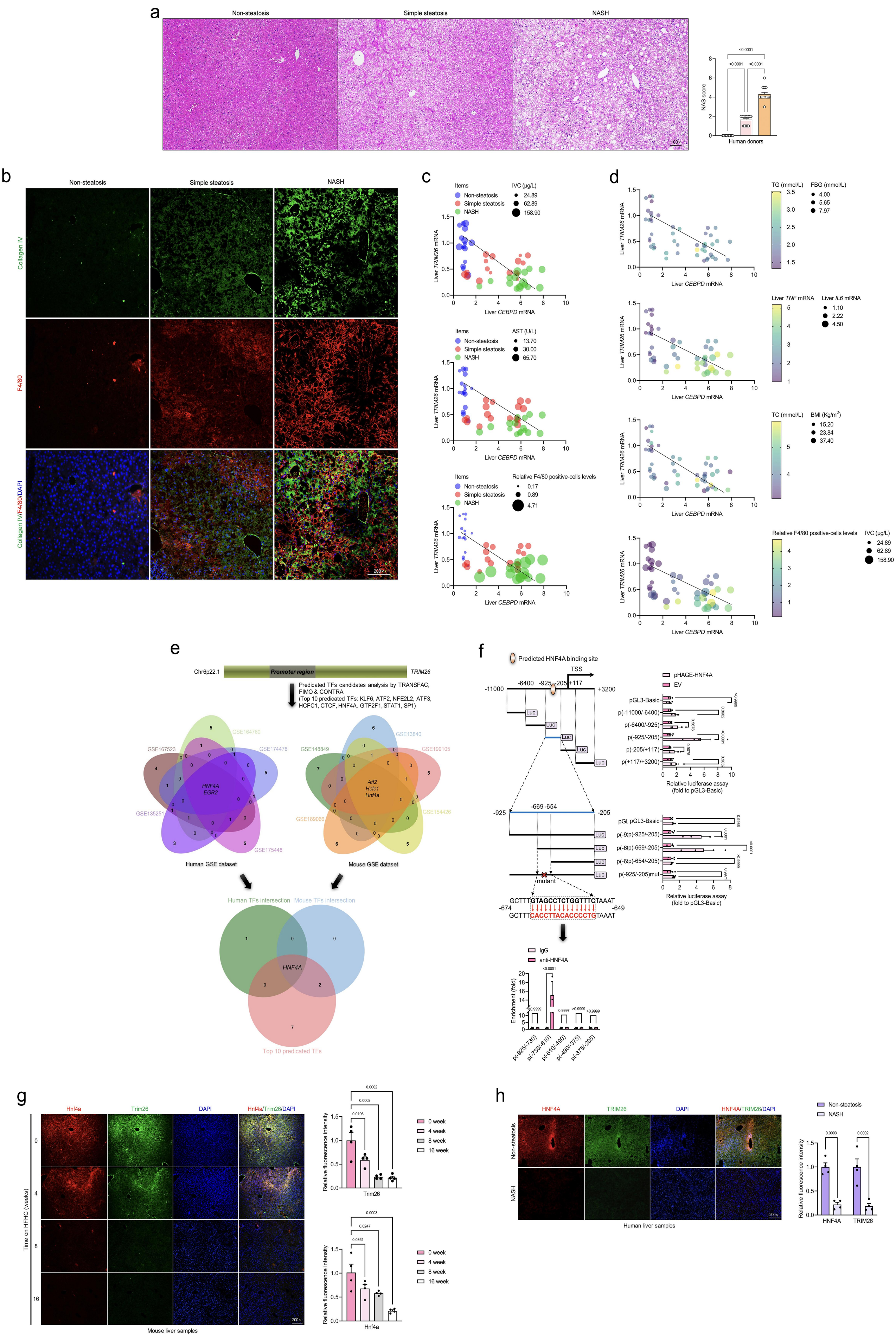

a
Non-steatosis
Simple steatosis
NASH
NAS score
Human donors
<0.0001
b
Non-steatosis
Simple steatosis
NASH
Collagen IV
F4/80
Collagen IV/F4/80/DAPI
c
Items
Non-steatosis
Simple steatosis
NASH
IVC (μg/L)
AST (U/L)
Relative F4/80 positive-cells levels
Liver TRIM26 mRNA
Liver CEBPD mRNA
d
TG (mmol/L)
FBG (mmol/L)
Liver TNF mRNA
Liver IL6 mRNA
TC (mmol/L)
BMI (Kg/m2)
Relative F4/80 positive-cells levels
IVC (μg/L)
Liver TRIM26 mRNA
Liver CEBPD mRNA
e
Chr6p22.1
Promoter region
TRIM26
Predicated TFs candidates analysis by TRANSFAC, FIMO & CONTRA
(Top 10 predicated TFs: KLF6, ATF2, NFE2L2, ATF3, HCFC1, CTCF, HNF4A, GTF2F1, STAT1, SP1)
GSE164760
GSE167523
GSE174478
GSE135251
GSE175448
HNF4A
EGR2
Human GSE dataset
GSE13840
GSE148849
GSE199105
GSE189066
GSE154426
Atf2
Hcfc1
Hnf4a
Mouse GSE dataset
Human TFs intersection
Mouse TFs intersection
HNF4A
Top 10 predicated TFs
f
Predicted HNF4A binding site
TSS
-11000
-6400
-925
-205
+117
+3200
Luc
pHAGE-HNF4A
EV
pGL3-Basic
p(-11000/-6400)
p(-6400/-925)
p(-925/-205)
p(-205/+117)
p(+117/+3200)
Relative luciferase assay
(fold to pGL3-Basic)
-669 -654
mutant
GCTTTGTAGCCTCTGGTTTCTAAAT
-674
-649
GCTTTCACCTTACACCCCTGTAAAT
p(-925/-205)mut
IgG
anti-HNF4A
Enrichment (fold)
p(-925/-730)
p(-730/-610)
p(-610/-490)
p(-490/-375)
p(-375/-205)
g
Hnf4a
Trim26
DAPI
Hnf4a/Trim26/DAPI
Time on HFHC (weeks)
Mouse liver samples
Relative fluorescence intensity
0 week
4 week
8 week
16 week
h
HNF4A
TRIM26
DAPI
HNF4A/TRIM26/DAPI
Non-steatosis
NASH
Human liver samples
Relative fluorescence intensity

Supplementary figure 1. TRIM26 expression is transcriptionally activated by HNF4A challenge with lipotoxicity.

(**a**) Representative H&E staining showing the histological change of liver samples from Non-steatosis, Simple steatosis and NASH patients, and corresponding NAS score (scale bars: magnification,100×, *n*=16 for Non-steatosis samples, *n*=17 for Simple steatosis, *n*=16 for NASH samples, *P*<0.01 by one-way ANOVA).

(**b**) Representative immunofluorescence images of Collagen IV and F4/80 expression in liver samples of Non-steatosis, Simple steatosis and NASH patients (scale bars: magnification, 200×).

(**c, d**) Correlation (c) and multiple Pearson correlations (d) analysis for the indicated indexes of clinical samples (*n*=16 for Non-steatosis samples, *n*=17 for Simple steatosis, *n*=16 for NASH samples, *P*<0.0001 by one-way ANOVA).

(**e**) Prediction of human TRIM26 promoter-binding transcription factors by TRANSFAC, FIMO & CONTRA software conjoint analysis and with the upstream 11,000 bp to downstream 3,200 bp region of TRIM26 gene transcription initiation site (TSS) set as the promoter region, with other parameters set at default. The lower venn diagrams showing the DEGs in human liver RNA-seq assay datasets with NAFLD or NASH phenotype (GSE167523, GSE164760, GSE174478, GSE175448 & GSE135251) and the HFD or HFHC group relative to corresponding controls from the mouse database in the RNA-seq assay (GSE148849, GSE13840, GSE199105, GSE154426 & GSE189066). DEGs defined from the pairwise comparisons were required to satisfy two selection criteria: a fold change >1.5 and adjusted *P* value <0.05.

(**f**) Relative luciferase activity in L02 cells of luciferase reporter constructs containing TRIM26 promoter truncations or its mutants transfected along with corresponding control plasmid pRL-TK followed by transfection with HNF4A or EV (empty vector). The relative luciferase activity is analyzed and then normalized to value determined in the pGL3-basic controls. The right bar graph showing the affinity of HNF4A on the TRIM26 promoter in HepG2 cells via chromatin immunoprecipitation (ChIP) assay using HNF4A antibody or IgG control, along with qPCR analysis with different primers (*n*=4 samples per group; *P*<0.0001 by 2 tailed *t* test).

(**g**) Representative immunofluorescence images of Trim26 and Hnf4a expression in lives of HFHC diet-fed WT mice over time (scale bars: magnification, 200×; *n*=4 images per group; *P*<0.05 by one-way ANOVA).

(**h**) Representative immunofluorescence images of TRIM26 and HNF4A expression in lives of human donors with non steatosis phenotypeand NASH phenotype (scale bars: magnification, 200×, *n* =4 images per group; *P*<0.001 by 2 tailed *t* test).

Data are expressed as mean ± SEM. The relevant experiments presented in this part were performed independently at least three times. *P*<0.05 indicates statistical significance.

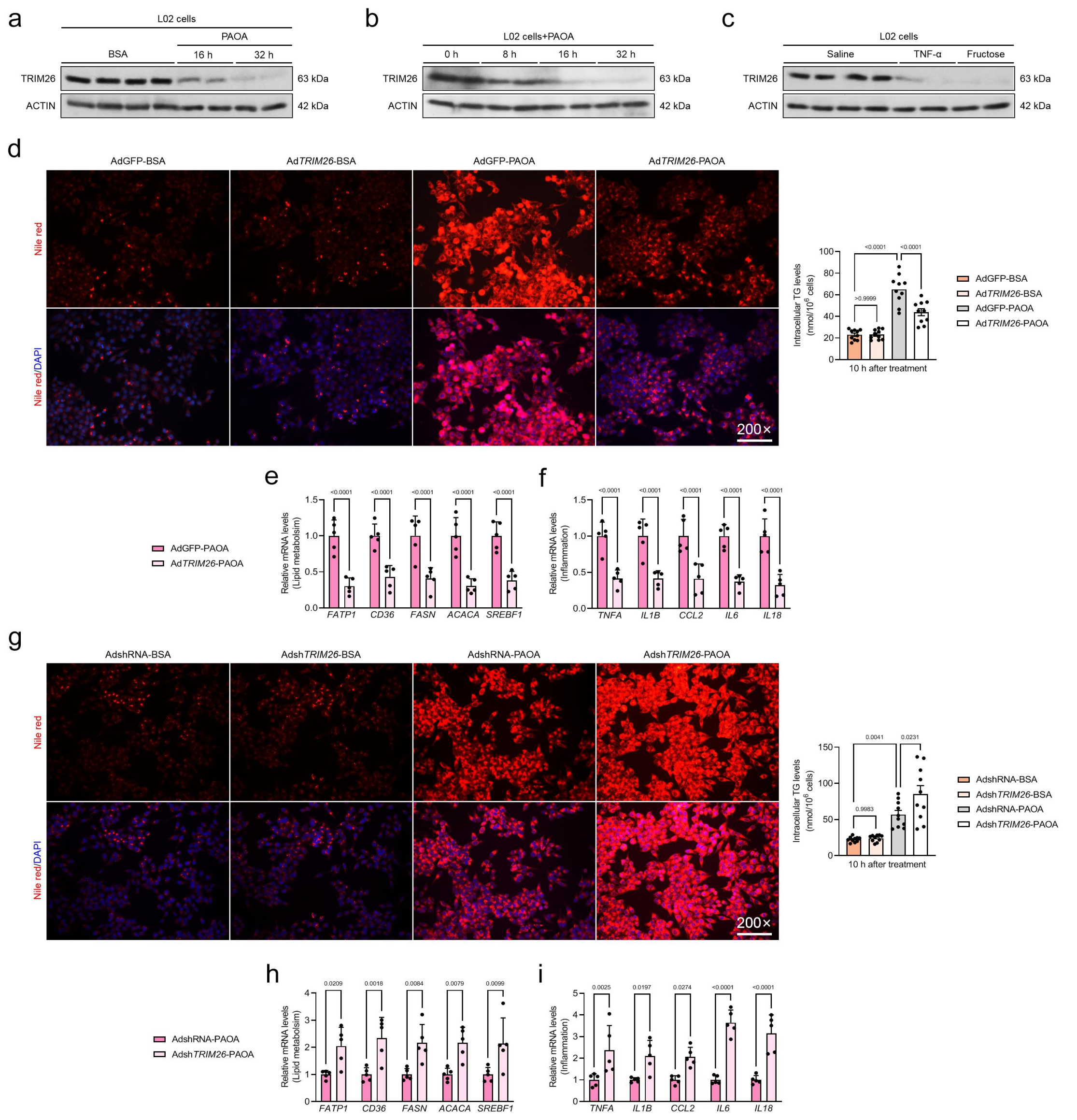

Supplementary figure 2. TRIM26 expression is downregulated in human hepatocytes upon challenge with metabolic insults.

(**a-c**) Representative immunoblotting bands of TRIM26 in the 0.5 mM palmitic acid+1.0 mM oleic acid (PAOA) mixture (a, b), 5 mM fructose or 100 ng/ml TNF-α (c)-induced WT L02 cells for time-course treatment or 32 hours treatment (n=4 per group).

(**d**) Representative images and intracellular triglyceride (TG) analysis of the Nile red staining of L02 cells that were transfected with Ad*TRIM26* and co-treated with PAOA for 10 hours (scale bars: magnification, 200×; n=10 images per group; P<0.05 by one-way ANOVA).

(**e, f**) qPCR analysis showing the lipid metabolism (e) and inflammation (f) related key genes expression changes in PAOA-treated Ad*TRIM26*-treasfected L02 cells (n=5 per group; P<0.001 by 2 tailed t test).

(**g**) Representative images and intracellular triglyceride (TG) analysis of the Nile red staining of L02 cells that were transfected with Adsh*TRIM26* and co-treated with PAOA for 10 hours (scale bars: magnification, 200×; n=10 images per group; P<0.05 by one-way ANOVA).

(**h, i**) qPCR analysis showing the lipid metabolism (h) and inflammation (i) related key genes expression changes in PAOA-treated Adsh*TRIM26*-treasfected L02 cells (n=5 per group; P<0.001 by 2 tailed t test).

Data are expressed as mean ± SEM. The relevant experiments presented in this part were performed independently at least three times. P <0.05 indicates statistical significance.

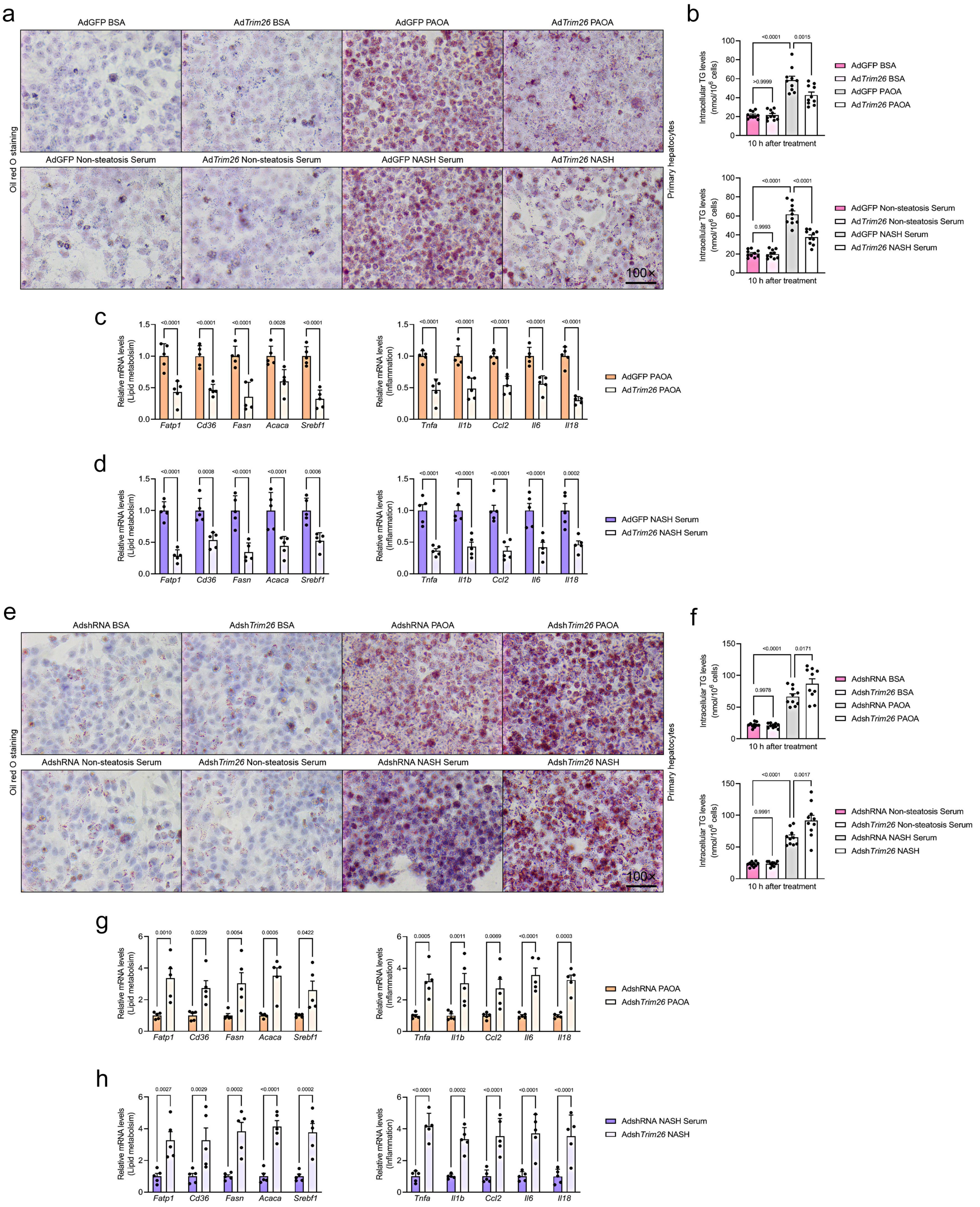

Supplementary figure 3. Trim26 restrains lipid deposition and inflammation in primary hepatocytes in response to NASH-like conditions.

(**a, b**) Representative images (a) and intracellular triglyceride (TG) (b) analysis of the oil red O staining of mice primary hepatocytes that were transfected with Ad*Trim26* and co-treated with PAOA or fresh culture medium containing NASH patients' serum for 10 hours (scale bars: magnification, 100×; *n*=10 images per group; *P*<0.01 by one-way ANOVA).

(**c, d**) qPCR analysis showing the lipid metabolism and inflammatory response related key genes expression alterations in PAOA (c)- or NASH serum (d)-treated Ad*Trim26*-treasfected primary hepatocytes (*n*=5 per group; *P*<0.001 by 2 tailed *t* test).

(**e, f**) Representative images (e) and intracellular triglyceride (TG) (f) analysis of the oil red O staining of mice primary hepatocytes that were transfected with Adsh*Trim26* and co-treated with PAOA or NASH serum for 10 hours (scale bars: magnification, 100×; *n*=10 images per group; *P*<0.05 by one-way ANOVA).

(**g, h**) qPCR analysis showing the lipid metabolism and inflammatory response related key genes expression alterations in PAOA (g)- or NASH serum (h)-treated Adsh*Trim26*-treasfected primary hepatocytes (*n*=5 per group; *P*<0.05 by 2 tailed *t* test).

Data are expressed as mean ± SEM. The relevant experiments presented in this part were performed independently at least three times. *P* <0.05 indicates statistical significance.

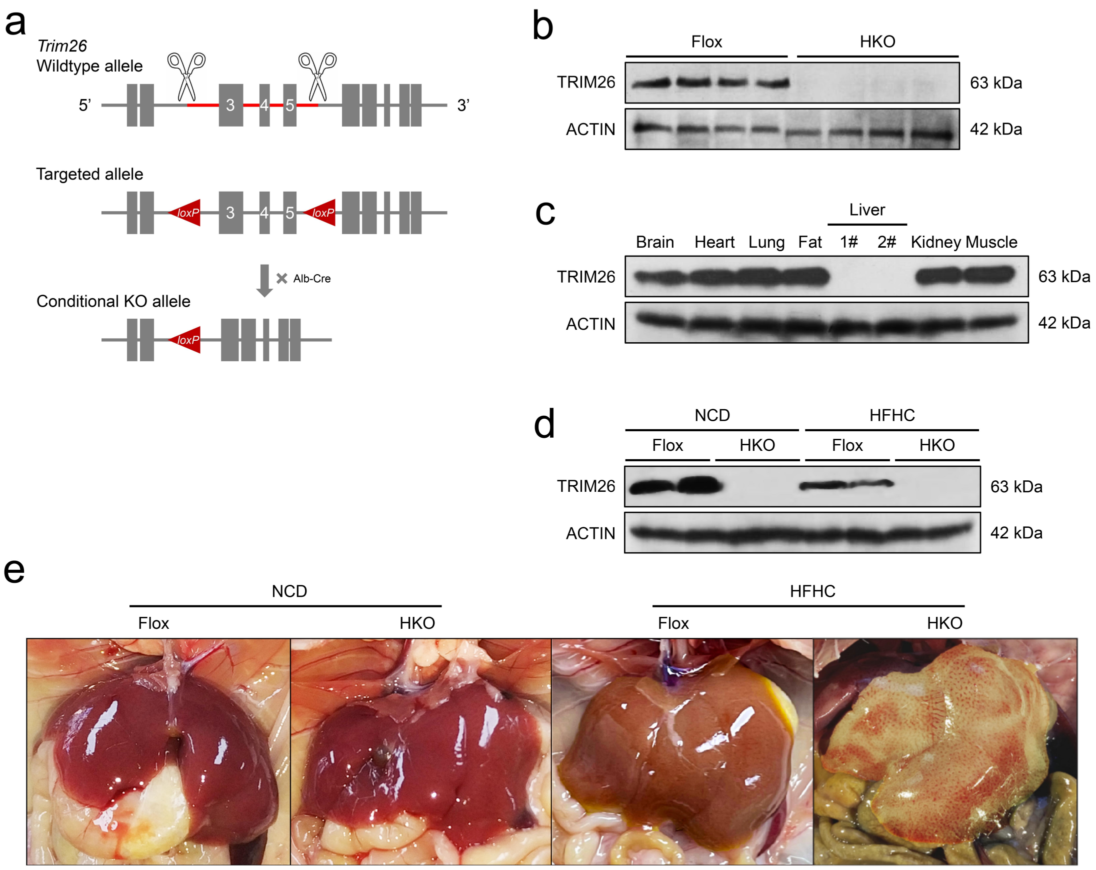

Supplementary figure 4. Establishment and experimental outline of hepatocyte-specific *Trim26* knockout mice.

(**a**) Experimental design for hepatocyte-specific *Trim26* knockout in rodent model.

(**b, c**) Characterization and representative immunoblotting bands of Trim26 expression levels in the liver samples of Flox mice and HKO mice (b), and in different tissue (c) of HKO mice (*n*=4 mice per group).

(**d**) Representative immunoblotting bands of Trim26 protein levels in liver samples of 16 weeks HFHC-fed Flox mice and HKO mice (*n*=4 mice per group).

(**e**) Representative liver appearance of the indicated groups.

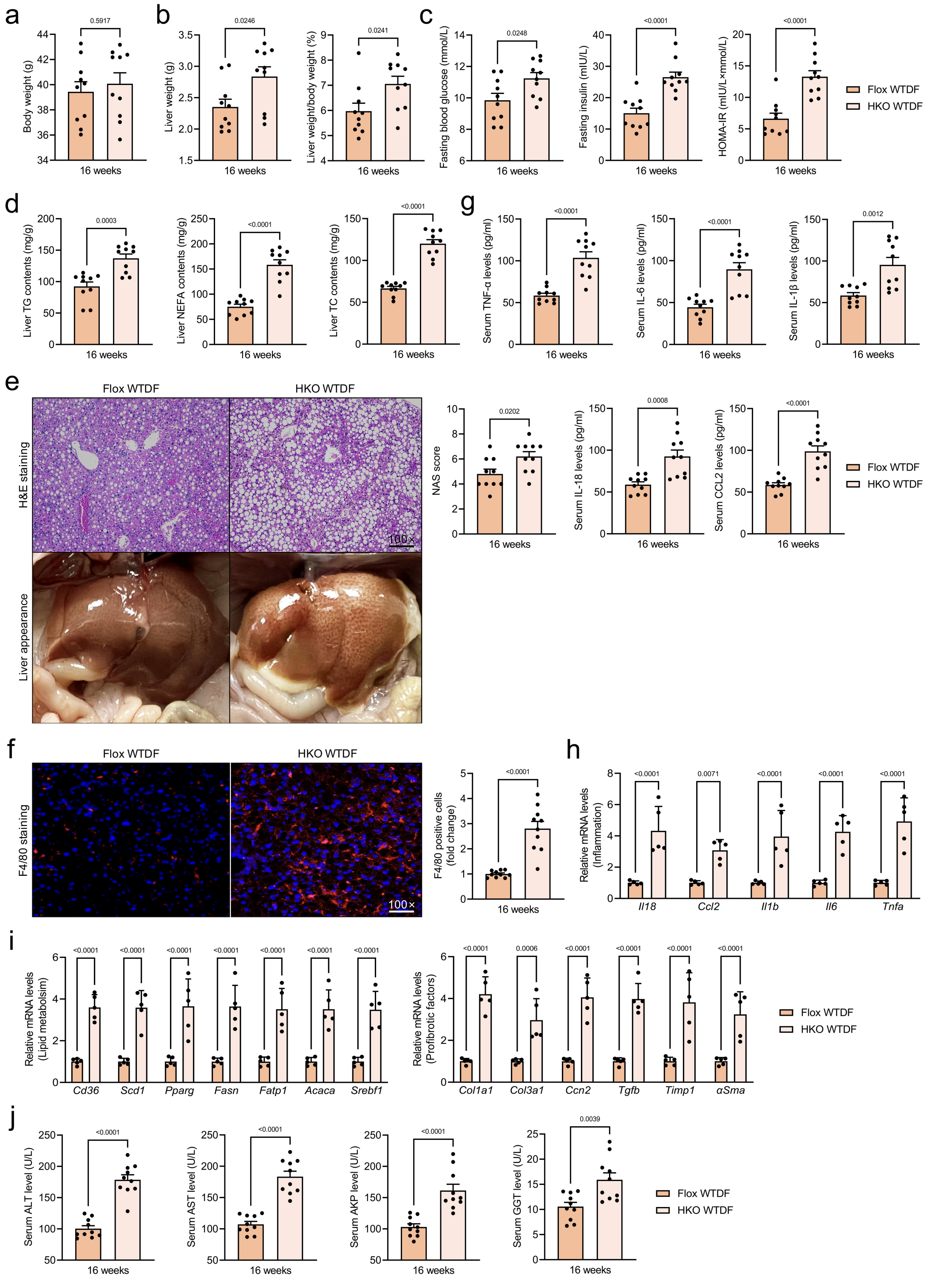

Supplementary figure 5. Hepatocyte-specific *Trim26* deficiency exacerbates WTDF-induced NASH pathologies.

(**a-d**) Records for the body weight (a), liver weight and the ratio of liver weight/body weight (%) (LW/BW) (b), fasting blood glucose levels, fasting insulin levels, HOMA-IR index (c), and liver TG, NEFA and TC contents (d) of the WTDF-fed floxed mice (Flox WTDF) and hepatocyte-specific *Trim26* ablation mice (HKO WTDF) (*n*=10 mice per group; *P*<0.05 by 2 tailed *t* test).

(**e, f**) Representative pictures of H&E staining and liver appearance, histological NAS score (e) changes and F4/80 expression (f) in WTDF-fed HKO or Flox mice (scale bars: magnification, 100×; *n*=10 images per group; *P*<0.05 by 2 tailed *t* test).

(**g, h**) Representative inflammation-related cytokines and genes expression profiles including TNF-α, IL-6, IL-1β, IL-18 and CCL2 in serum (*n*=10 mice per group) (g) or livers (h) from WTDF-fed HKO or Flox mice (*n*=5 mice per group; *P*<0.05 by 2 tailed *t* test).
(**i**) qPCR analysis showing the lipid metabolism- and profibrosis-related genes expression profiles in livers from WTDF-fed HKO or Flox mice (*n*=5 mice per group; *P*<0.01 by 2 tailed *t* test).
(**j**) Representative liver function-related indicators including ALT, AST, AKP and GGT in serum from WTDF-fed HKO or Flox mice (*n*=10 mice per group; *P*<0.01 by 2 tailed *t* test).
Data are expressed as mean ± SEM. The relevant experiments presented in this part were performed independently at least three times. *P* <0.05 indicates statistical significance.

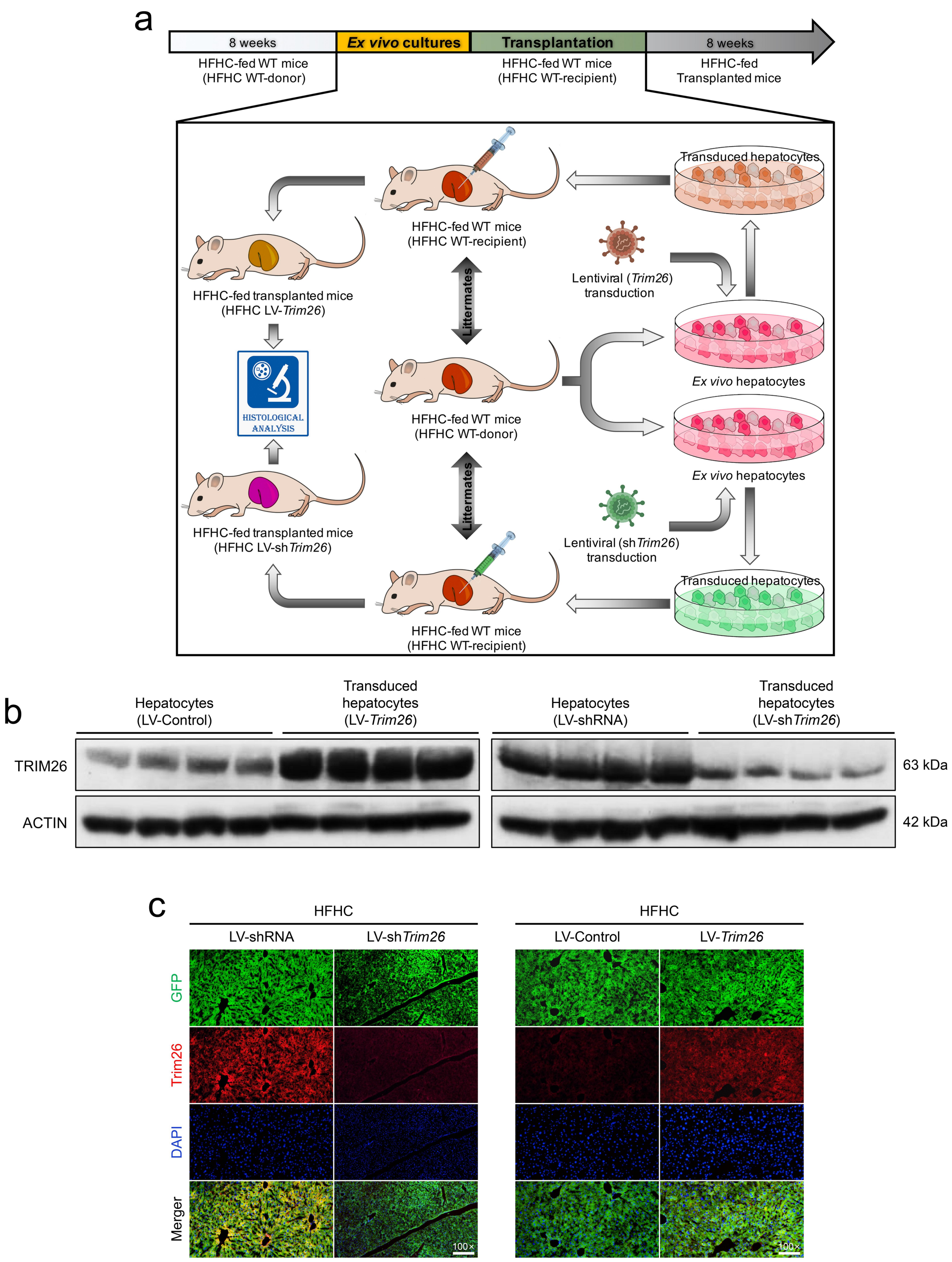

Supplementary figure 6. Establishment and experimental outline of allogeneic hepatocyte transplantation using lentivirus (LV)-mediated expression alterations of Trim26.

(**a**) Schematic diagram of experimental design for the *ex vivo*-mediated gene therapy. Primary hepatocytes from the preconditioned WT mice with an 8-weeks HFHC treatment as donor were isolated and *ex vivo* cultured. The cultured hepatocytes were transduced with lentivirus-loaded *Trim26* or sh*Trim26* sequences. The corresponding blank vectors were transduced as controls. Then, the additional HFHC-fed littermates mice as recipient were injected with transduced hepatocytes via portal vein. The HFHC-fed transplanted mice (HFHC LV-*Trim26* or HFHC LV-sh*Trim26*) were then fed with HFHC diet for additional 8 weeks.

(**b**) Representative western blotting bands showing the lentivirus (LV)-mediated overexpression or inhibition of Trim26 protein expression in transduced hepatocytes (n=8 images per group, n=8

were analyzed in total but only 4 are shown).

(**c**) Characterization of transfection efficiency of lentivirus (LV)-triggered overexpression or inhibition of Trim26 in hepatocytes of mice after 16 weeks HFHC diet administration (scale bars: magnification, 100×) (*n*=8 images per group).

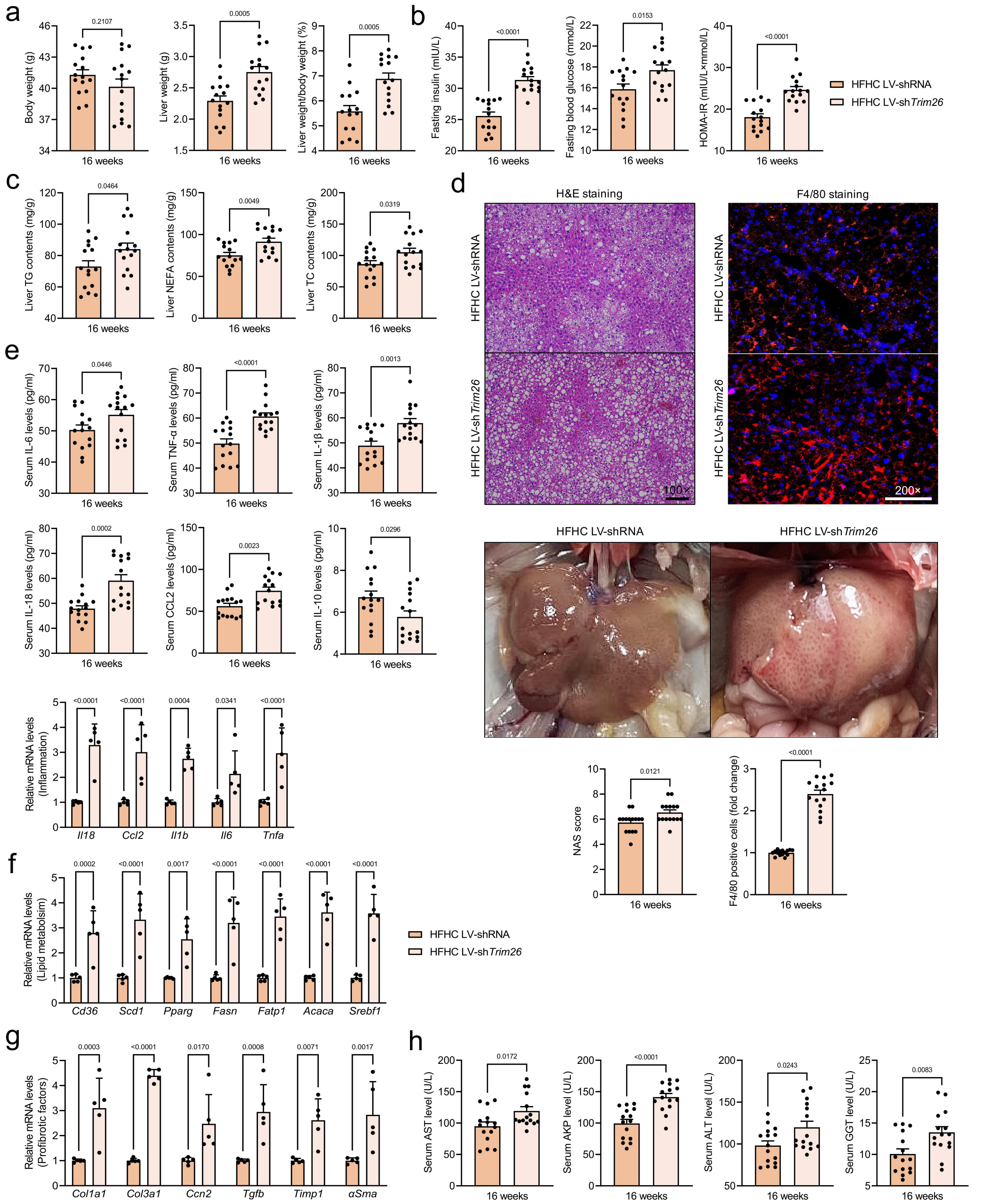

Supplementary figure 7. Allogeneic hepatocyte transplantation using decreased Trim26 aggravates NASH diet-induced liver metabolism dysregulation.

(**a-c**) Records for the body weight, liver weight and the ratio of liver weight/body weight (%) (a), fasting insulin levels, fasting blood glucose levels and HOMA-IR index (b), and liver TG, NEFA and TC contents (c) of the HFHC LV-sh*Trim26* mice at the last week of NASH diet treatment (*n*=15 mice per group; *P*<0.05 by 2 tailed *t* test).

(**d**) Representative pictures of H&E staining (magnification, 100×; *n*=15 images per group), F4/80 staining (scale bars: magnification, 200×; *n*=15 images per group; *P*<0.05 by 2 tailed *t* test) and liver appearance in the HFHC LV-sh*Trim26* mice after *ex vivo* experiment.

(**e**) Records for serum IL-6, TNF-α, IL-1β, IL-18, CCL2 and IL-10 (*n*=15 mice per group; *P*<0.05 by 2 tailed *t* test) and liver inflammation-related gene expression changes (*n*=5 samples per group; *P*<0.05 by 2 tailed *t* test) in HFHC LV-sh*Trim26* mice after *ex vivo* experiment.

(**f, g**) qPCR analysis showing lipid metabolism (f)- and profibrotic factors (g)-related gene

expression changes in HFHC LV-sh*Trim26* mice after *ex vivo* experiment (*n*=5 samples per group; *P*<0.05 by 2 tailed *t* test).

(**h**) Records for serum AST, AKP, ALT and GGT contents in HFHC LV-sh*Trim26* mice after *ex vivo* experiment (*n*=15 samples per group; *P*<0.05 by 2 tailed *t* test).

Data are expressed as mean ± SEM. The relevant experiments presented in this part were performed independently at least three times. Significance determined by Student's 2-tailed *t*-test analysis. *P* <0.05 indicates statistical significance.

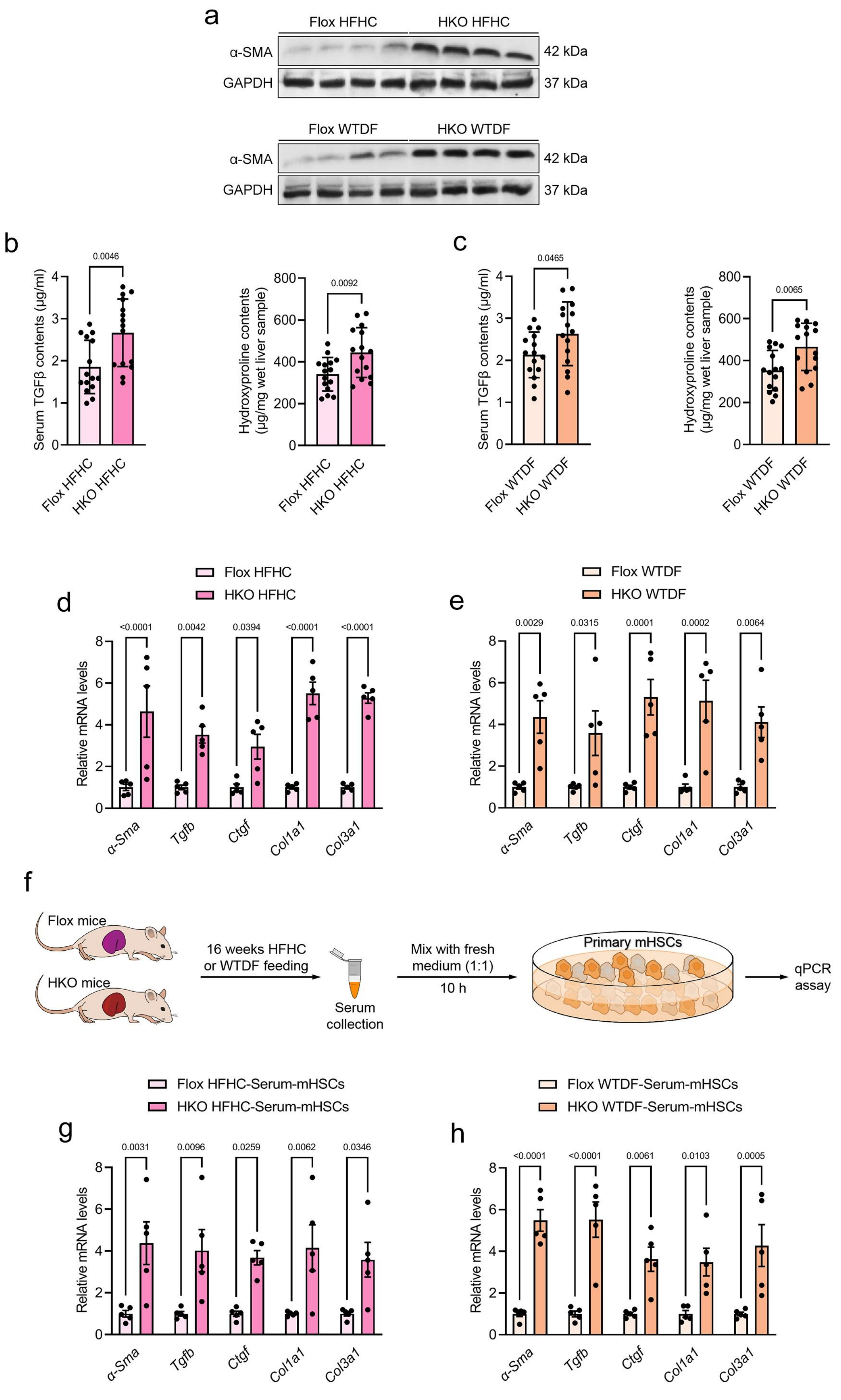

a
Flox HFHC
HKO HFHC
α-SMA
42 kDa
GAPDH
37 kDa
Flox WTDF
HKO WTDF
α-SMA
42 kDa
GAPDH
37 kDa
b
0.0046
Serum TGFβ contents (μg/ml)
Flox HFHC
HKO HFHC
0.0092
Hydroxyproline contents (μg/mg wet liver sample)
Flox HFHC
HKO HFHC
c
0.0465
Serum TGFβ contents (μg/ml)
Flox WTDF
HKO WTDF
0.0065
Hydroxyproline contents (μg/mg wet liver sample)
Flox WTDF
HKO WTDF
d
Flox HFHC
HKO HFHC
<0.0001
0.0042
0.0394
<0.0001
<0.0001
Relative mRNA levels
α-Sma
Tgfb
Ctgf
Col1a1
Col3a1
e
Flox WTDF
HKO WTDF
0.0029
0.0315
0.0001
0.0002
0.0064
Relative mRNA levels
α-Sma
Tgfb
Ctgf
Col1a1
Col3a1
f
Flox mice
HKO mice
16 weeks HFHC or WTDF feeding
Serum collection
Mix with fresh medium (1:1)
10 h
Primary mHSCs
qPCR assay
g
Flox HFHC-Serum-mHSCs
HKO HFHC-Serum-mHSCs
0.0031
0.0096
0.0259
0.0062
0.0346
Relative mRNA levels
α-Sma
Tgfb
Ctgf
Col1a1
Col3a1
h
Flox WTDF-Serum-mHSCs
HKO WTDF-Serum-mHSCs
<0.0001
<0.0001
0.0061
0.0103
0.0005
Relative mRNA levels
α-Sma
Tgfb
Ctgf
Col1a1
Col3a1

Supplementary figure 8. Functional loss of hepatocyte *Trim26* accelerates HFHC/WTDF-triggered NASH-associated fibrosis pathologies.

(**a**) Representative western blotting bands showing α-Sma protein expression changes in liver tissue from the functional loss of hepatocyte *Trim26* mice that were treated with 16-weeks HFHC or WTDF diet (n=4 samples per group).

(**b, c**) Determination of serum TGFβ levels or liver hydroxyproline contents in HKO HFHC and Flox HFHC mice (b), and HKO WTDF and Flox WTDF mice (c) (n=15 per group; P<0.05 by 2 tailed t test).

(**d, e**) qPCR analysis showing fibrosis-related indexes including *α-Sma*, *Tgfb*, *Ctgf*, *Col1a1* and *Col3a1* levels in liver samples collected from HKO HFHC and Flox HFHC mice (d), and HKO WTDF and Flox WTDF mice (e) (n=5 per group; P<0.05 by 2 tailed t test).

(**f**) A simple outline indicating pretreatment method of primary mHSCs prior to qPCR analysis. After 16-weeks HFHC/WTDF diet-administration, the serum of Flox mice and HKO mice were collected. The harvested serum was mixed with fresh medium at 1:1 ratio. Then, mHSCs were coincubated with the mixed medium for 10 h.

(**g, h**) qPCR analysis showing fibrosis-related indexes including *α-Sma*, *Tgfb*, *Ctgf*, *Col1a1* and *Col3a1* levels in post-treated mHSCs during HFHC serum (g) or WTDF serum (h) administration (n=5 per group; P<0.05 by 2 tailed t test).

Data are expressed as mean ± SEM. The relevant experiments in this section were carried out independently at least three times. P <0.05 indicates statistical significance.

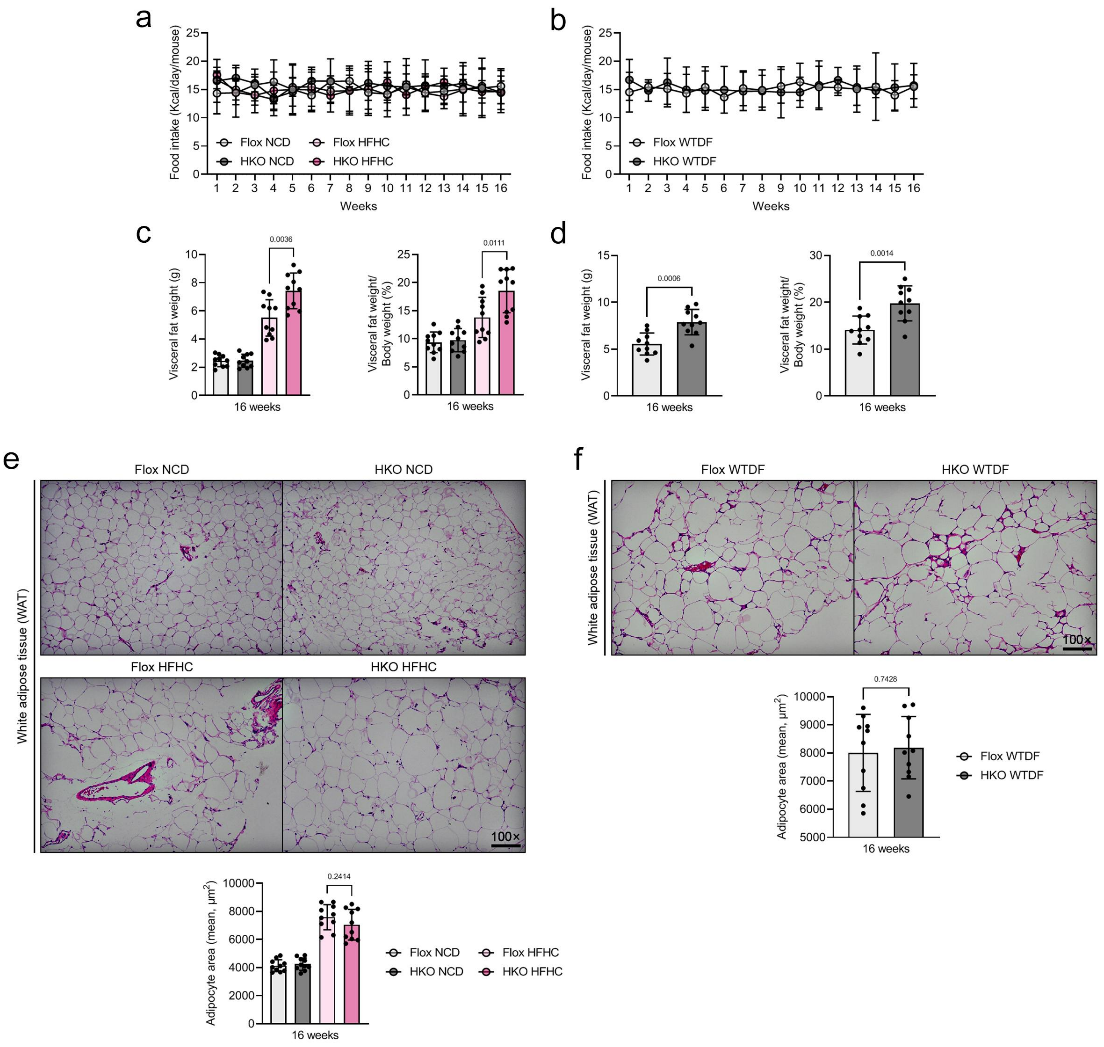

Supplementary figure 9. Functional loss of hepatocyte *Trim26* accelerates weight gain of adipose tissue to promote HFHC/WTDF-induced NASH pathologies.

(**a**) Records for the food intake (Kcal/day/per mouse) of assigned group in a 16-weeks HFHC or NCD treatment (n=10 mice per group; P<0.05 by 2 tailed t test).

(**b**) Records for the food intake (Kcal/day/per mouse) of assigned group in a 16-weeks WTDF treatment (n=10 mice per group; P<0.05 by 2 tailed t test).

(**c**) Visceral fat weight and visceral fat weight/body weight ratio of the assigned group after 16-weeks HFHC or NCD treatment (n=10 mice per group; P<0.05 by 2 tailed t test).

(**d**) Visceral fat weight and visceral fat weight/body weight ratio of the assigned group after 16-weeks WTDF treatment (n=10 mice per group; P<0.05 by 2 tailed t test).

(**e**) The representative H&E staining images and the corresponding analyzed adipocytes area (mean, μm^2) on the white adipose tissue (WAT) sections of mice in the assigned group (scale bars: magnification, 100×; n=10 images per group; P<0.05 by 2 tailed t test).

(**f**) The representative H&E staining images and the corresponding analyzed adipocytes area (mean, μm^2) on the white adipose tissue (WAT) sections of mice in the assigned group (scale bars: magnification, 100×; n=10 images per group; P<0.05 by 2 tailed t test).

Data are expressed as mean ± SEM. The relevant experiments in this section were carried out independently at least three times. P <0.05 indicates statistical significance.

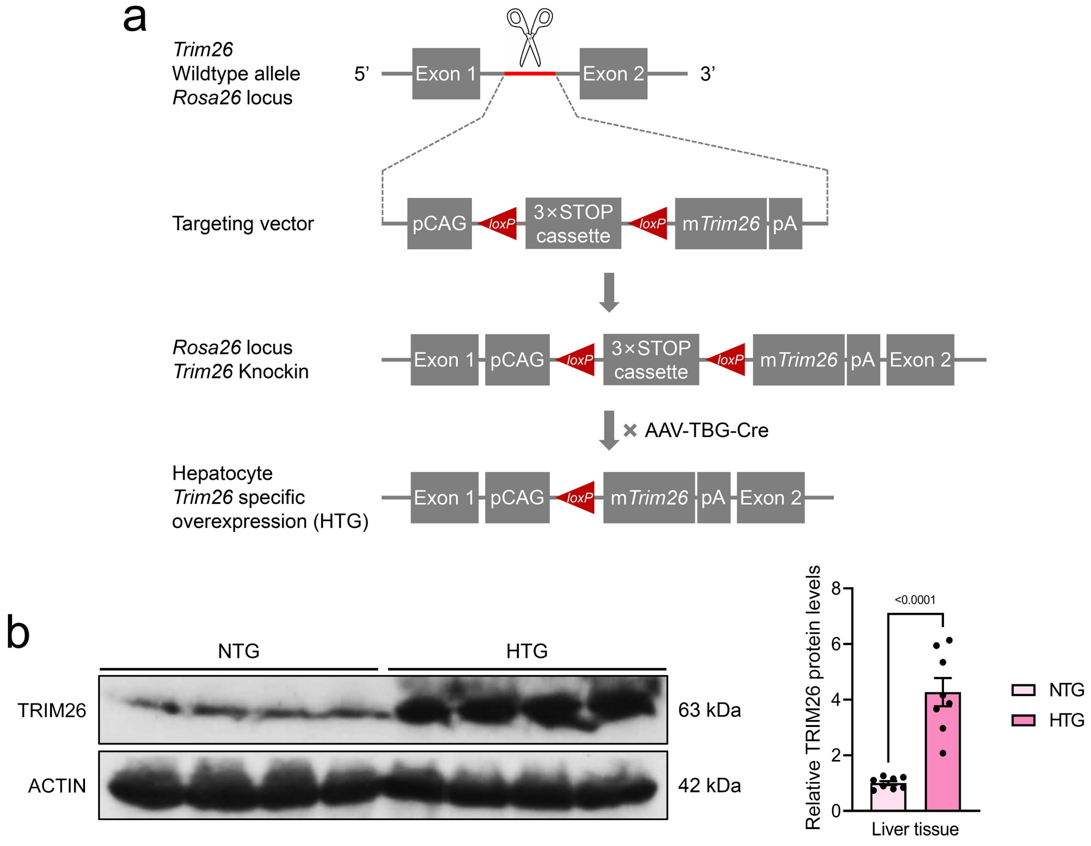

Supplementary figure 10. Establishment and experimental outline of hepatocyte-specific *Trim26* knockin mice.

(**a**) Schematic diagram of the establishment of hepatocyte-specific *Trim26* overexpression (HTG) mouse strain.

(**b**) Characterization and representative western blotting bands of TRIM26 expression in the liver samples of control mice (NTG) and HTG mice (n=8 mice per group; P<0.0001 by 2 tailed t test). Data are expressed as mean ± SEM. The relevant experiments presented in this part were performed independently at least three times. P <0.05 indicates statistical significance.

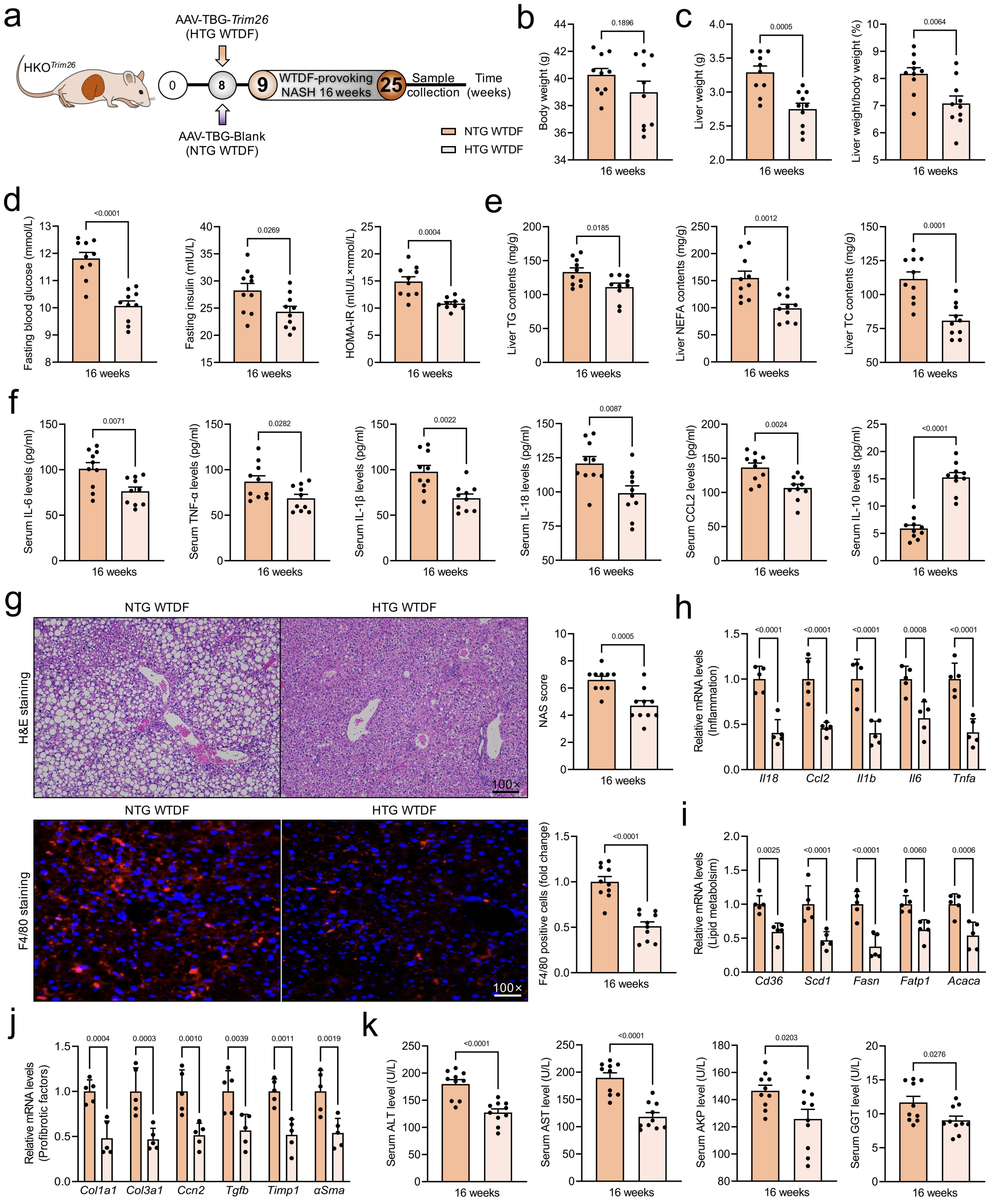

Supplementary figure 11. Hepatocyte-specific *Trim26* restoration ameliorates WTDF-induced NASH pathologies.

(**a**) Schematic plot of adeno-associated virus (serotype 8)-TBG-Cre (AAV-TBG-Cre)-mediated *Trim26* restoration in liver of WTDF-fed HKO^{Trim26} mice (HTG WTDF). The AAV-TBG-Blank was used as control (NTG WTDF).

(**b-e**) Records for the body weight (b), liver weight and the ratio of liver weight/body weight (%) (c), fasting blood glucose levels, fasting insulin levels and HOMA-IR index (d) and liver lipid contents including TG, NEFA and TC (e) in the HTG WTDF and NTG WTDF mice (n=10 mice per group; P<0.05 by 2 tailed t test).

(**f**) Representative inflammation-related cytokines and genes expression profiles including IL-6, TNF-α, IL-1β, IL-18, CCL2 and IL-10 in serum from WTDF-fed HTG or NTG mice (n=10 mice per group; P<0.05 by 2 tailed t test).

(**g**) Representative pictures of H&E staining, histological NAS score and F4/80 staining in HTG WTDF and NTG WTDF mice (scale bars: magnification, 100×; n=10 images per group; P<0. 05 by 2 tailed t test).

(**h-j**) Representative mRNA levels of inflammation (h)-, lipid metabolism (i)-, and profibrosis (j)-related genes expression in livers from WTDF-fed HTG or NTG mice (n=5 mice per group; P<0.05 by 2 tailed t test).
(**k**) Representative liver function-related indicators including ALT, AST, AKP and GGT in serum from WTDF-fed HTG or NTG mice (n=10 mice per group; P<0.05 by 2 tailed t test).
Data are expressed as mean ± SEM. The relevant experiments presented in this part were performed independently at least three times. P <0.05 indicates statistical significance.

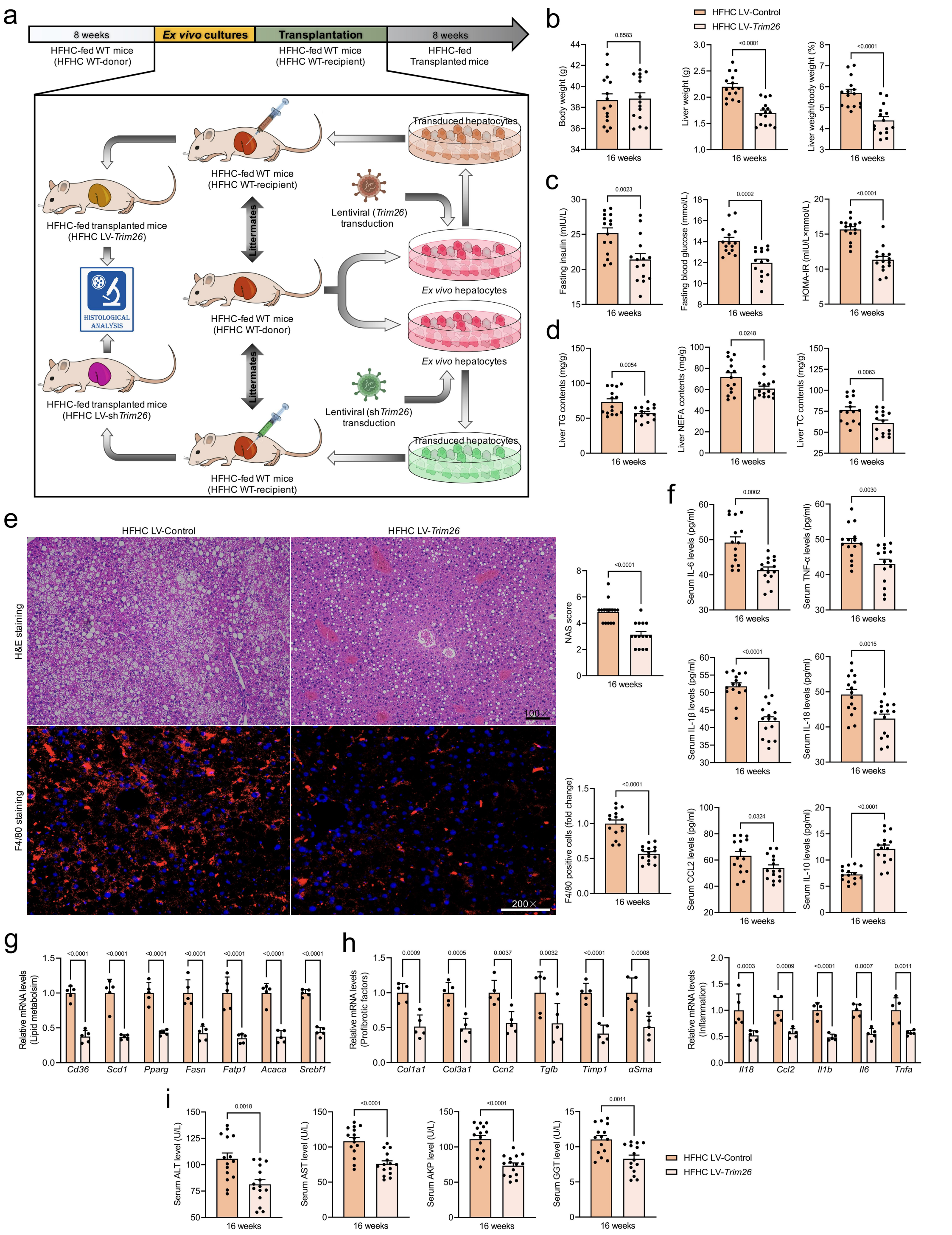

Supplementary figure 12. Allogeneic hepatocyte transplantation using increased Trim26 assuages NASH diet-induced liver metabolism dysregulation.

(**a**) Experimental design of allogeneic hepatocyte transplantation.

(**b-d**) Records for the body weight, liver weight and the ratio of liver weight/body weight (%) (b), fasting insulin levels, fasting blood glucose levels and HOMA-IR index (c) and liver lipid contents (d) including TG, TC and NEFA of the HFHC LV-*Trim26* mice (n=15 mice per group; P<0.05 by 2 tailed t test).

(**e**) Representative pictures of H&E staining (scale bars: magnification, 100×) and F4/80 staining (scale bars: magnification, 200×) and corresponding histological NAS score in the livers of HFHC LV-*Trim26* mice after *ex vivo* experiment (*n*=15 images per group; *P*<0.05 by 2 tailed *t* test).
(**f**) Representative inflammation-related cytokines including TNF-α, IL-1β, IL-6, CCL2, IL-18 and IL-10 in serum from HFHC LV-*Trim26* mice and controls (*n*=15 mice per group; *P*<0.05 by 2 tailed *t* test).
(**g, h**) qPCR analysis indicating the lipid metabolism (g)-, profibrotic factor- and inflammation (h)-related genes expression alteration in liver of HFHC LV-*Trim26* mice after *ex vivo* experiment (*n*=5 per group; *P*<0.05 by 2 tailed *t* test).
(**i**) Representative liver function-related indicators including AST, ALT, AKP and GGT in serum from HFHC LV-*Trim26* mice and controls (*n*=15 mice per group; *P*<0.05 by 2 tailed *t* test).
Data are expressed as mean±SEM. The relevant experiments presented in this part were performed independently at least three times. *P* <0.05 indicates statistical significance.

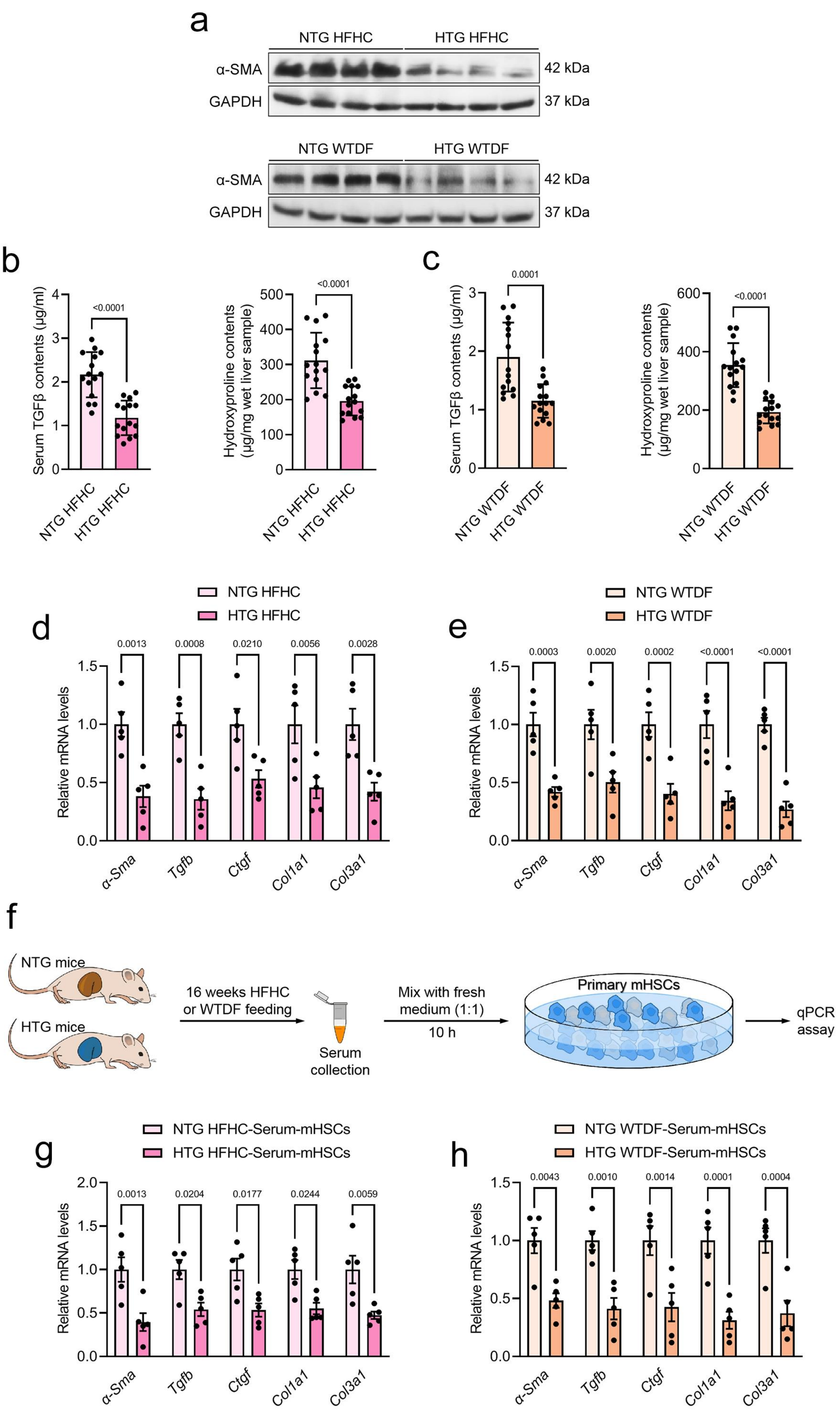
a
NTG HFHC
HTG HFHC
α-SMA
42 kDa
GAPDH
37 kDa
NTG WTDF
HTG WTDF
α-SMA
42 kDa
GAPDH
37 kDa
b
Serum TGFβ contents (μg/ml)
<0.0001
Hydroxyproline contents (μg/mg wet liver sample)
<0.0001
NTG HFHC
HTG HFHC
c
Serum TGFβ contents (μg/ml)
0.0001
Hydroxyproline contents (μg/mg wet liver sample)
<0.0001
NTG WTDF
HTG WTDF
d
NTG HFHC
HTG HFHC
Relative mRNA levels
0.0013
0.0008
0.0210
0.0056
0.0028
α-Sma
Tgfb
Ctgf
Col1a1
Col3a1
e
NTG WTDF
HTG WTDF
Relative mRNA levels
0.0003
0.0020
0.0002
<0.0001
<0.0001
α-Sma
Tgfb
Ctgf
Col1a1
Col3a1
f
NTG mice
HTG mice
16 weeks HFHC or WTDF feeding
Serum collection
Mix with fresh medium (1:1)
10 h
Primary mHSCs
qPCR assay
g
NTG HFHC-Serum-mHSCs
HTG HFHC-Serum-mHSCs
Relative mRNA levels
0.0013
0.0204
0.0177
0.0244
0.0059
α-Sma
Tgfb
Ctgf
Col1a1
Col3a1
h
NTG WTDF-Serum-mHSCs
HTG WTDF-Serum-mHSCs
Relative mRNA levels
0.0043
0.0010
0.0014
0.0001
0.0004
α-Sma
Tgfb
Ctgf
Col1a1
Col3a1

Supplementary figure 13. Functional restoration of hepatocyte *Trim26* mitigates HFHC/WTDF-induced NASH-associated fibrosis pathologies.

(**a**) Representative western blotting bands showing α-Sma protein expression alterations in liver samples from the functional restoration of hepatocyte *Trim26* mice that were treated with 16-weeks HFHC or WTDF diet (*n*=4 samples per group).

(**b, c**) Determination of serum TGFβ levels or liver hydroxyproline contents in HTG HFHC and NTG HFHC mice (B), and HTG WTDF and NTG WTDF mice (C) (*n*=15 per group; *P*<0.05 by 2 tailed *t* test).

(**d, e**) qPCR analysis showing fibrosis-related indexes including *α-Sma*, *Tgfb*, *Ctgf*, *Col1a1* and *Col3a1* levels in liver samples collected from HTG HFHC and NTG HFHC mice (D), and HTG WTDF and NTG WTDF mice (E) (*n*=5 per group; *P*<0.05 by 2 tailed *t* test).

(**f**) A simple outline indicating pretreatment method of primary mHSCs prior to qPCR analysis. After 16-weeks HFHC/WTDF diet-administration, the serum of NTG mice and HTG mice were collected. The harvested serum was mixed with fresh medium at 1:1 ratio. Then, mHSCs were coincubated with the mixed medium for 10 h.

(**g, h**) qPCR analysis showing fibrosis-related indexes including *α-Sma*, *Tgfb*, *Ctgf*, *Col1a1* and *Col3a1* levels in post-treated mHSCs (*n*=5 per group; *P*<0.05 by 2 tailed *t* test).

Data are expressed as mean ± SEM. The relevant experiments in this section were carried out independently at least three times. *P* <0.05 indicates statistical significance.

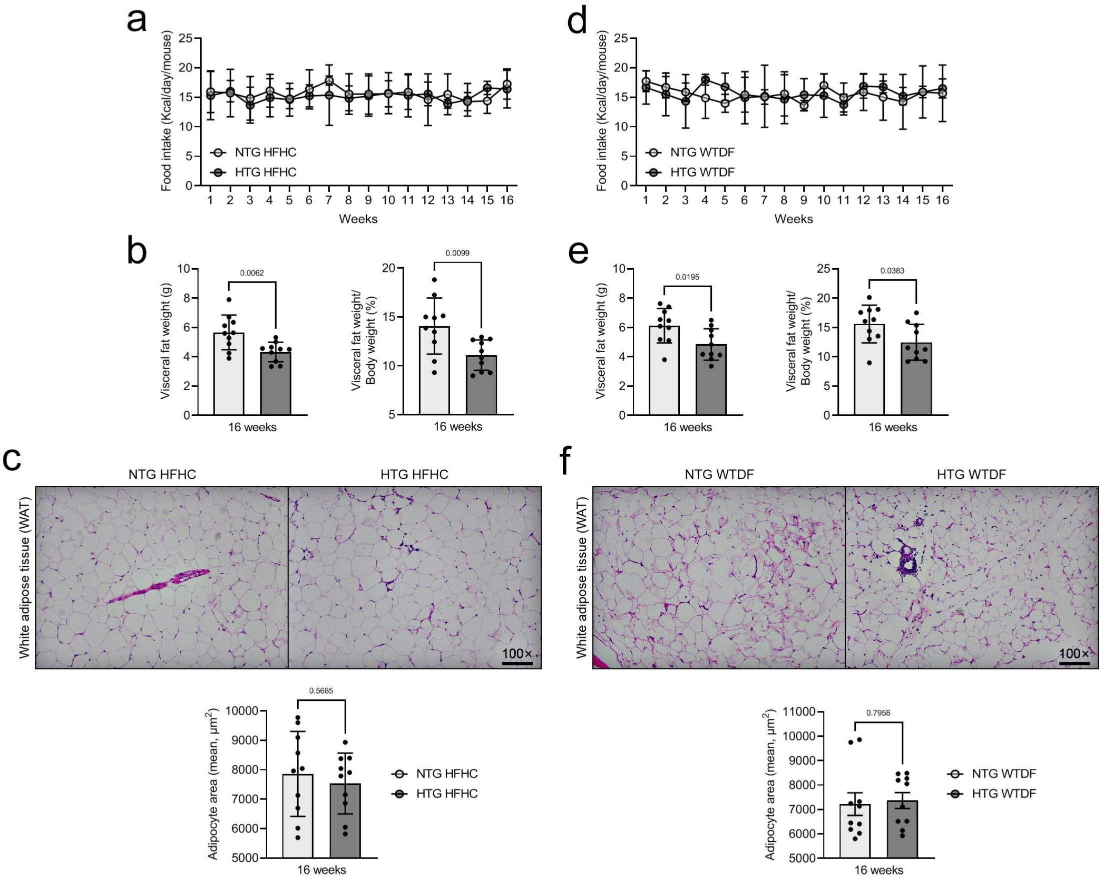

Supplementary figure 14. Functional restoration of hepatocyte *Trim26* decreases weight gain of adipose tissue to mitigate HFHC/WTDF-induced NASH pathologies.

(**a**) Records for the food intake (Kcal/day/per mouse) of assigned group in a 16-weeks HFHC treatment (n=10 mice per group; P<0.05 by 2 tailed t test).

(**b**) Visceral fat weight and visceral fat weight/body weight ratio of the assigned group after 16-weeks HFHC treatment (n=10 mice per group; P<0.05 by 2 tailed t test).

(**c**) The representative H&E staining images and the corresponding analyzed adipocytes area (mean, μm^2) on the white adipose tissue (WAT) sections of mice in the assigned group (scale bars: magnification, 100×; n=10 images per group; P<0.05 by 2 tailed t test).

(**d**) Records for the food intake (Kcal/day/per mice) of assigned group in 16-weeks WTDF treatment (n=10 mice per group; P<0.05 by 2 tailed t test).

(**e**) Visceral fat weights and visceral fat weight/body weight ratio of the indicated mice, after 16-weeks WTDF treatment (n=10 mice per group; P<0.05 by 2 tailed t test).

(**f**) The representative H&E staining pictures and the analyzed adipocytes area (mean, μm^2) on the white adipose tissue (WAT) sections of mice in the assigned group (scale bars: magnification, 100×; n=10 images per group; P<0.05 by 2 tailed t test).

Data are expressed as mean ± SEM. The relevant experiments in this section were carried out independently at least three times. P <0.05 indicates statistical significance.

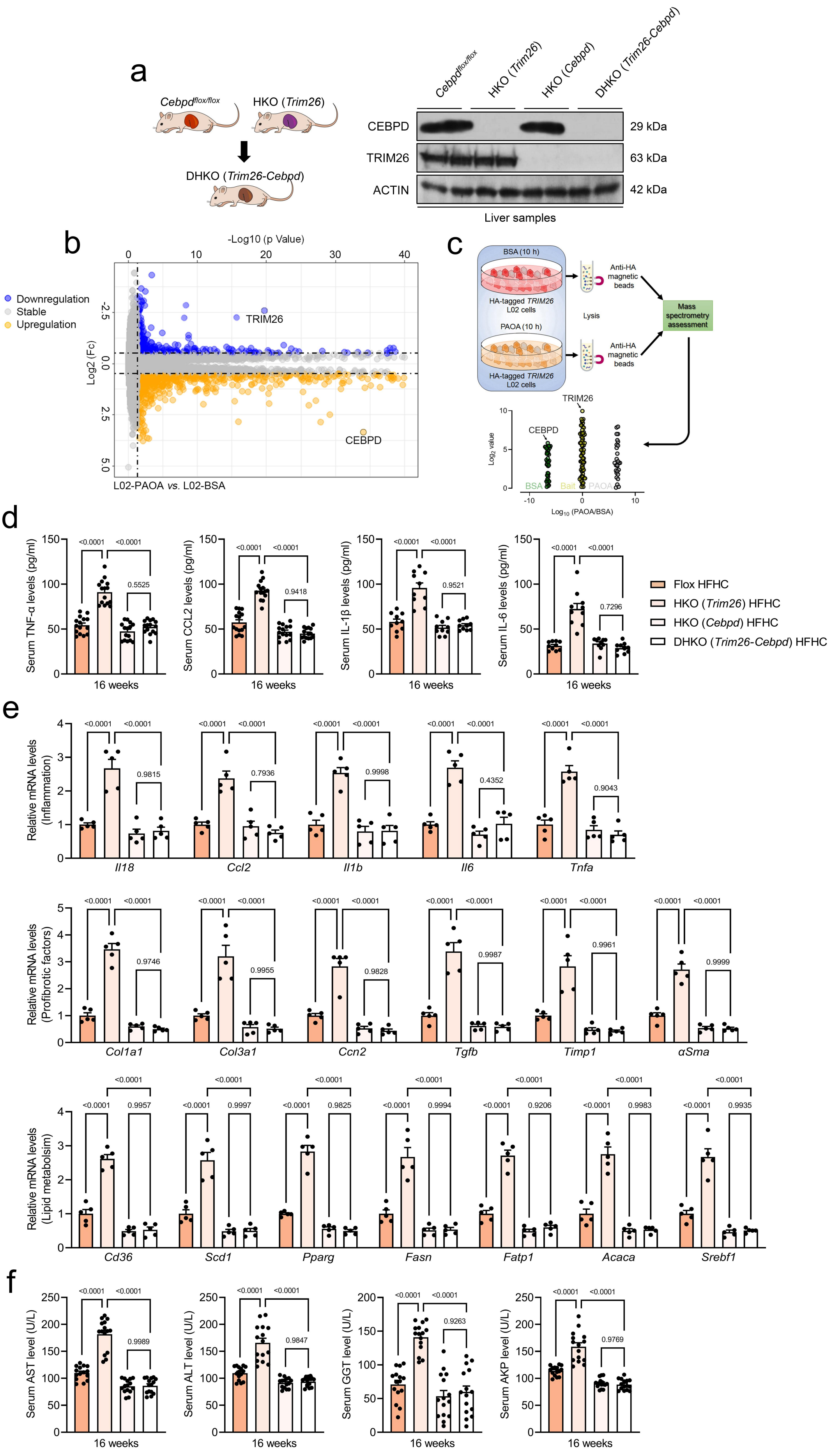

Supplementary figure 15. Cebpd signaling is required for the protective function of Trim26 against NASH diet-induced hepatic inflammation, lipid accumulation and abnormal liver function.

(**a**) Schematic diagram of the establishment of hepatocyte-specific *Cebpd* and *Trim26* double knockout, DHKO (*Cebpd*-*Trim26*) mouse strain by crossing *Cebpd*$^{flox/flox}$ mice with HKO (*Trim26*) mice (left). Characterization and representative western blotting bands of Cebpd and Trim26 expression in the livers of *Cebpd*$^{flox/flox}$, HKO (*Trim26*), HKO (*Cebpd*) and DHKO (*Cebpd*-*Trim26*) mice (n=4 mice per group) (right).

(**b**) Volcano plot showing genes expression variation in L02 cells after PAOA treatment.

(**c**) Schematic of the experimental procedure used to identify potential target of TRIM26. Scatter diagram showing the TRIM26-interacting proteins in L02 cells after co-treatment of PAOA or BSA; each point represents a protein. Green, proteins identified only in BSA-treated cells; Yellow, proteins identified in bait-treated cells; Gray, proteins identified only in PAOA-treated cells.

(**d**) Records for serum TNF-α, CCL2, IL-1β and IL-6 contents in indicated groups in HKO (*Trim26*) HFHC, HKO (*Cebpd*) HFHC, DHKO (*Trim26*-*Cebpd*) HFHC and Flox HFHC group (n=10-15 mice per group; P<0.0001 by one-way ANOVA).

(**e**) qPCR analysis showing the inflammation-, pro-fibrosis- and lipid metabolism-related gene expression changes in livers of HKO (*Trim26*) HFHC, HKO (*Cebpd*) HFHC, DHKO (*Trim26*-*Cebpd*) HFHC and Flox HFHC group (n=5 mice per group; P<0.0001 by one-way ANOVA).

(**f**) Records for serum liver function-related indicators including AST, ALT, GGT and AKP contents in HKO (*Trim26*) HFHC, HKO (*Cebpd*) HFHC, DHKO (*Trim26*-*Cebpd*) HFHC and Flox HFHC group (n=15 mice per group; P<0.0001 by one-way ANOVA).

Data are expressed as mean±SEM. The relevant experiments presented in this part were performed independently at least three times. P <0.05 indicates statistical significance.

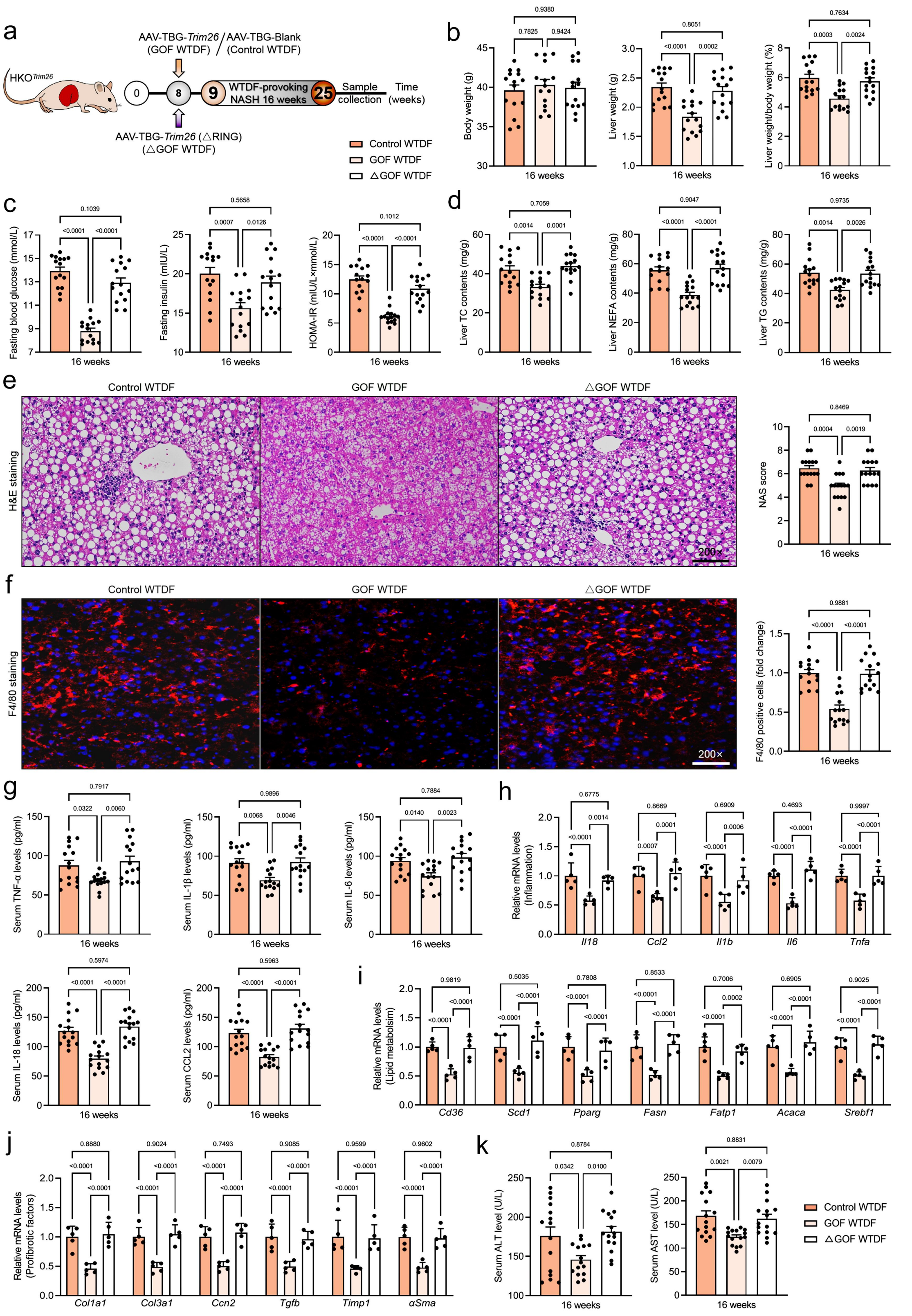

Supplementary figure 16. Mutational *Trim26* (△RING) cannot effectively alleviate WTDF-induced dysregulated liver metabolism.

(**a**) Schematic diagram of adeno-associated virus (serotype 8)-TBG-Cre (AAV-TBG-Cre)-mediated *Trim26* restoration (GOF WTDF) or *Trim26* (△RING) restoration (△GOF WTDF) in liver of WTDF-fed HKO^{Trim26} mice. The AAV-TBG-Blank was used as control (Control WTDF).

(**b-d**) Records for the body weight, liver weight and the ratio of liver weight/body weight (%) (b), fasting blood glucose levels, fasting insulin levels and HOMA-IR index (c), and liver lipid contents including TC, NEFA and TG (d) of the indicated mice (*n*=15 mice per group; *P*<0.05 by one-way ANOVA).
(**e, f**) Representative pictures of H&E staining (e), F4/80 staining (f) and corresponding NAS score showing the hepatosteatosis, inflammatory infiltration and liver injury levels in the indicated mice (scale bars: magnification, 200×; *n*=15 mice per group; *P*<0.05 by one-way ANOVA).
(**g**) Representative inflammation-related cytokines contents including TNF-α, IL-1β, IL-6, IL-18 and CCL2 in serum from WTDF-fed indicated mice (*n*=15 mice per group; *P*<0.05 by one-way ANOVA).
(**h-j**) qPCR analysis showing the mRNA levels alterations in inflammation (h)-, lipid metabolism (i)- and fibrosis (j)-associated key genes in livers of WTDF-fed indicated mice (*n*=5 mice per group; *P*<0.05 by one-way ANOVA).
(**k**) Representative liver function-related indicators including ALT and AST in serum from WTDF-fed indicated mice (*n*=15 mice per group; *P*<0.05 by one-way ANOVA).
Data are expressed as mean ± SEM. The relevant experiments presented in this part were performed independently at least three times. *P* <0.05 indicates statistical significance.

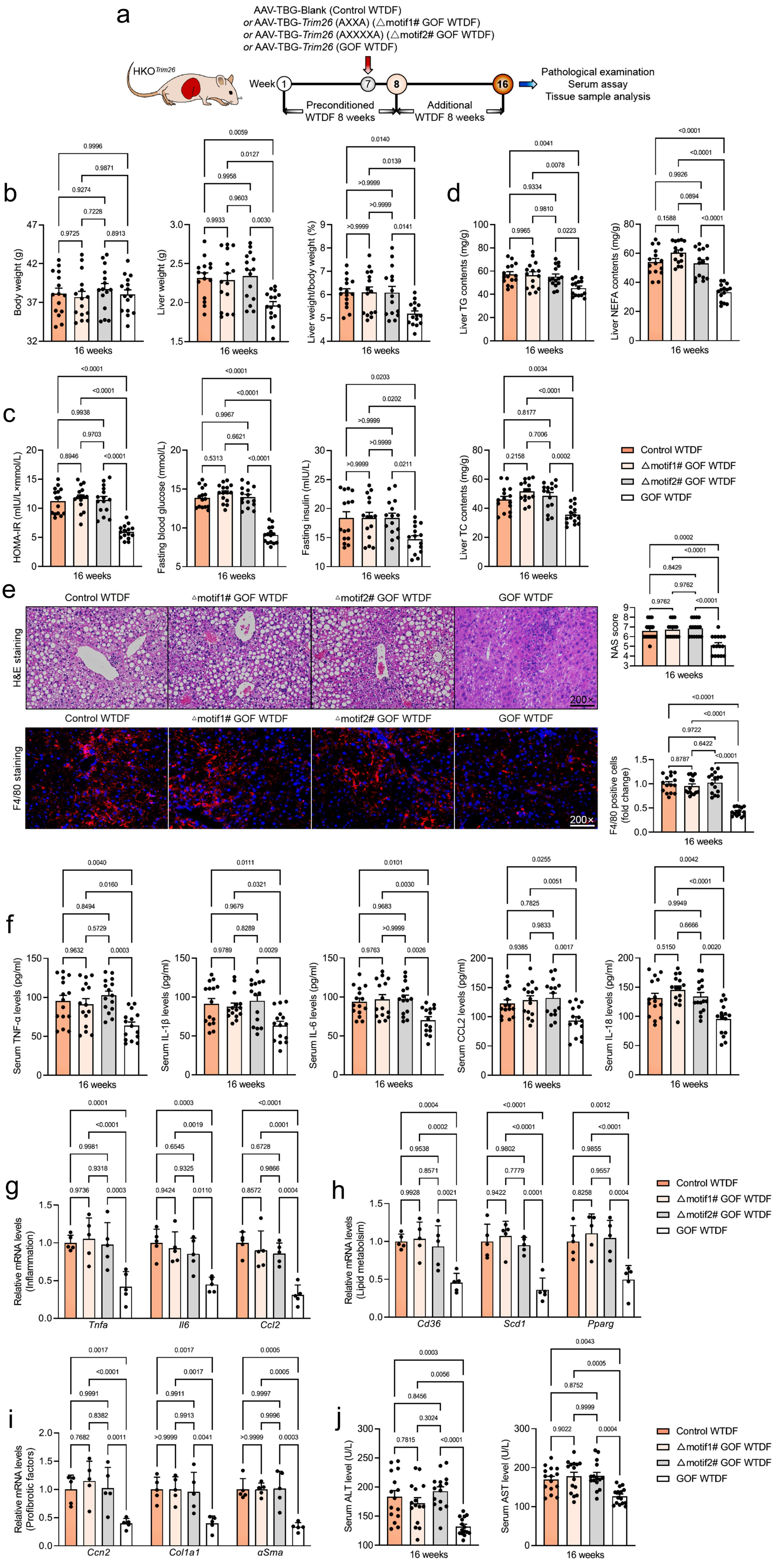
a
AAV-TBG-Blank (Control WTDF)
or AAV-TBG-Trim26 (AXXA) (△motif1# GOF WTDF)
or AAV-TBG-Trim26 (AXXXXA) (△motif2# GOF WTDF)
or AAV-TBG-Trim26 (GOF WTDF)
HKO Trim26
Week 1 7 8 16
Preconditioned WTDF 8 weeks
Additional WTDF 8 weeks
Pathological examination
Serum assay
Tissue sample analysis
b
Body weight (g)
Liver weight (g)
Liver weight/body weight (%)
16 weeks
d
Liver TG contents (mg/g)
Liver NEFA contents (mg/g)
c
HOMA-IR (mIU/L×mmol/L)
Fasting blood glucose (mmol/L)
Fasting insulin (mIU/L)
Liver TC contents (mg/g)
Control WTDF
△motif1# GOF WTDF
△motif2# GOF WTDF
GOF WTDF
e
H&E staining
F4/80 staining
200×
NAS score
F4/80 positive cells (fold change)
f
Serum TNF-α levels (pg/ml)
Serum IL-1β levels (pg/ml)
Serum IL-6 levels (pg/ml)
Serum CCL2 levels (pg/ml)
Serum IL-18 levels (pg/ml)
g
Relative mRNA levels (Inflammation)
Tnfa
Il6
Ccl2
h
Relative mRNA levels (Lipid metabolism)
Cd36
Scd1
Pparg
i
Relative mRNA levels (Profibrotic factors)
Ccn2
Col1a1
αSma
j
Serum ALT level (U/L)
Serum AST level (U/L)

Supplementary figure 17. *Trim26* with motif#1 (AXXA) and motif#2 (AXXXXA) mutants in RING domain fail to mitigate WTDF-triggered abnormal liver metabolism.

(**a**) Schematic diagram of adeno-associated virus (serotype 8)-TBG-Cre (AAV-TBG-Cre)-mediated *Trim26* restoration (GOF WTDF), *Trim26* (AXXA) restoration (△motif#1 GOF WTDF) or *Trim26* (AXXXXA) restoration (△motif#2 GOF WTDF) in liver of WTDF-fed HKO^{Trim26} mice. The AAV-TBG-Blank was used as control (Control WTDF).

(**b-d**) Records for the body weight, liver weight and the ratio of liver weight/body weight (%) (b), fasting blood glucose levels, fasting insulin levels and HOMA-IR index (c), and liver TG, NEFA and TC contents (d) in the indicated mice (n=15 mice per group; P<0.05 by one-way ANOVA).

(**e**) Representative pictures of H&E staining, F4/80 staining and corresponding NAS score showing the hepatosteatosis, inflammatory infiltration and liver injury levels in the indicated mice (scale bars: magnification, 200×; n=15 mice per group; P<0.05 by one-way ANOVA).

(**f**) Representative inflammation-related cytokines contents including TNF-α, IL-1β, IL-6, IL-18 and CCL2 in serum from WTDF-fed indicated mice (n=15 mice per group; P<0.05 by one-way ANOVA).

(**g-i**) qPCR analysis showing the mRNA levels alterations in inflammation (g)-, lipid metabolism (h)- and fibrosis (i)-associated key genes in livers of WTDF-fed indicated mice (n=5 mice per group; P<0.05 by one-way ANOVA).

(**j**) Representative liver function-related indicators including ALT and AST in serum from WTDF-fed indicated mice (n=15 mice per group; P<0.05 by one-way ANOVA).

Data are expressed as mean ± SEM. The relevant experiments presented in this part were performed independently at least three times. P <0.05 indicates statistical significance.

Supplementary table 1. The clinical information of non-steatosis, simple steatosis and NASH patients.

Sex	Male/Female (11/5)	Male/Female (10/7)	Male/Female (8/8)
Age (y)	35.06±8.00	35.70±8.54	35.93±10.94
BMI (Kg/m^2)	19.55±2.47 [a, b]	25.10±1.32 [b]	30.04±2.84
Serum AST (U/L)	24.79±7.53 [a, b]	54.18±8.48	51.93±9.52
Serum ALT (U/L)	22.21±8.46 [a, b]	45.50±7.62	42.06±8.67
Serum TG (mmol/L)	1.71±0.28 [a, b]	2.13±0.24 [b]	2.27±0.42
Serum TC (mmol/L)	3.76±0.48 [a, b]	4.17±0.43 [b]	4.42±0.85
Serum FBG (mmol/L)	5.03±0.75 [a, b]	5.71±0.70 [b]	6.27±0.77
Liver *IL6* mRNA	1.55±0.24 [a, b]	2.45±0.27 [b]	3.48±0.57
Liver *TRIM26* mRNA	0.95±0.30 [a, b]	0.52±0.17 [b]	0.27±0.13
Liver *TNF* mRNA	1.39±0.31 [a, b]	2.37±0.39 [b]	4.45±0.39
Liver *CEBPD* mRNA	0.95±0.26 [a, b]	3.99±1.93 [b]	5.76±1.41
Serum LN (μg/L)	78.27±28.65 [a, b]	92.56±25.89 [b]	153.84±40.36
Serum HA (μg/L)	36.97±11.24 [b]	31.95±13.51 [b]	105.06±26.23
Serum IVC (μg/L)	70.93±25.75 [b]	71.01±30.76 [b]	111.39±26.72
Serum PCIII (μg/L)	49.25±22.90 [a, b]	52.84±24.95 [b]	120.00±41.23
Serum GGT (U/L)	28.91±10.82 [a, b]	36.68±18.61 [b]	68.39±16.52
Serum AKP (U/L)	86.69±19.32 [a, b]	106.84±30.55 [b]	181.33±37.51
NAS score	0.00±0.00 [a, b]	1.64±0.49 [b]	4.31±0.70

These data are expressed as the mean ± SEM. [a]P<0.05 versus the Simple steatosis donors; [b] P<0.05 versus the NASH donors.

Abbreviation: BMI, Body mass index; AST, Aspartate transaminase; ALT, Alanine aminotransferase; TG, Triglyceride; TC, Total cholesterol; FBG, Fasting blood glucose; IL6, interleukin-6; TRIM26, Tripartite motif containing 26; TNF, Tumor necrosis factor; CEBPD, CCAAT/enhancer binding protein delta; LN, Laminin; HA, hyaluronidase; IVC, Collagen Type IV; PCIII, Type III procollagen; GGT, γ-glutamyl transpeptidase; AKP, Alkline phosphatase

Supplementary table 2. The primer sequences for the targeted genes used in this study.

Primers	Forward Sequence (5’-3’)	Reverse Sequence (5’-3’)
TRIM26	TGCACTACTACTGTGAGGACG	TCCTTAGGGTACTCAGGTGGT
TNFα	ACCTGGCCTCTCTACCTTGT	CCCGTAGGGCGATTACAGTC
IL-1β	AGCAAAGTGATAGGCCGAC	GCGTCGCAATTAGTGTTGTG
IL-6	GGGCAACAAGATTCCGATATA	AGCCACTACATGGAATCTAAT
CCL2	CTGGTCCGAGTGAGACAAAG	AGATCAGGCTCTGATGGAGAA
IL-8	ATGACTTCCAAGCTGGCCGTGGCT	TCTCAGCCCTCTTCAAAAACTTCTC
IL-18	CAAGGCTGGTCCATGCTCC	TGCTATCACTTCCTTTCTGTTGC
CD36	AGCAACTGGTGGATGGTTTC	TCAAGGGAGAGCACTGGTTT
SCD1	TCAAGGGAGAGCACTGGTTT	TGAATGTTCTTGTCGTAGGG
PPARG	CTATGGAGTTCATGCTTGTG	GTACTGACATTTATTT
FASN	CTGCGGAAACTTCAGGAAATG	GGTTCGGAATGCTATCCAGG
FATP1	GATGTGCTCTACGACTGTCTG	CAGCCCATAGATGAGACACTG
ACACA	GGCCAGTGCTATGCTGAGAT	AGGGTCAAGTGCTGCTCCA
SREBF1	CGGCGCTGCTGACCGACATC	CCCTGCCCCACTCCCAGCAT
COL1A1	GATTCCCTGGACCTAAAGGTGC	AGCCTCTCCATCTTTGCCAGCA
COL3A1	TGGTCTGCAAGGAATGCCTGGA	TCTTTCCCTGGGACACCATCAG
CCN2	CTTGCGAAGCTGACCTGGAAGA	CCGTCGGTACATACTCCACAGA
TGFB	TACCTGAACCCGTGTTGCTCTC	GTTGCTGAGGTATCGCCAGGAA
TIMP1	GGAGAGTGTCTGCGGATACTTC	GCAGGTAGTGATGTGCAAGAGTC
αSMA	CTGTTCCAGCCATCCTTCAT	TCATGATGCTGTTGTAGGTGGT
GAPDH	TGCACCACCAACTGCTTAGC	GGCATGGACTGTGGTCATGAG

Abbreviation: TRIM26, Tripartite motif containing 26; TNFα, Tumor necrosis factor α; IL-1β, Interleukin 1 Beta; IL-6, Interleukin 6; CCL2, C-C motif chemokine ligand 2; IL-8, Interleukin 8; IL-18, Interleukin 18; CD36, Cluster determinant 36; SCD1, Stearoyl-CoA desaturase 1; PPARG, Peroxisome proliferator activated receptor gamma; FASN, Fatty acid synthase; FATP1, Fatty acid transport protein 1, ACACA, Acetyl-CoA carboxylase alpha; SREBF1, Sterol regulatory element binding transcription factor 1; COL1A1, Collagen type I alpha 1 chain; COL3A1, Collagen type III alpha 1 chain; CCN2, Cellular communication network factor 2; TGFB, Transforming growth factor beta 1; TIMP1, Tissue inhibitor of metalloproteinases 1; αSMA, α-smooth muscle actin; GAPDH, Glyceraldehyde-3-phosphate dehydrogenase

Supplementary table 3. Small guide RNA (sgRNA) for human TRIM26 gene.

TRIM26	oligo F	ACCGTGTGGCAACTGGCCAGCCTGG
TRIM26	oligo R	AAACCCAGGCTGGCCAGTTGCCACA
