# Supplementary material for: Tripartite motif containing 26 prevents steatohepatitis progression by suppressing C/EBPδ signalling activation
Source: Nat Commun. 2023 Oct 11;14:6384. doi: 10.1038/s41467-023-42040-9 (PMC10567751; doi:10.1038/s41467-023-42040-9)
Supplement: Supplementary file 2 — Reporting Summary [file 41467_2023_42040_MOESM2_ESM.pdf]

nature portfolio

Corresponding author(s): Minxuan Xu

Last updated by author(s): Aug 9, 2023

Reporting Summary

Nature Portfolio wishes to improve the reproducibility of the work that we publish. This form provides structure for consistency and transparency in reporting. For further information on Nature Portfolio policies, see our Editorial Policies and the Editorial Policy Checklist.

Statistics

For all statistical analyses, confirm that the following items are present in the figure legend, table legend, main text, or Methods section.

n/a	Confirmed	
☐	☒	The exact sample size (*n*) for each experimental group/condition, given as a discrete number and unit of measurement
☐	☒	A statement on whether measurements were taken from distinct samples or whether the same sample was measured repeatedly
☐	☒	The statistical test(s) used AND whether they are one- or two-sided<br>*Only common tests should be described solely by name; describe more complex techniques in the Methods section.*
☐	☒	A description of all covariates tested
☐	☒	A description of any assumptions or corrections, such as tests of normality and adjustment for multiple comparisons
☐	☒	A full description of the statistical parameters including central tendency (e.g. means) or other basic estimates (e.g. regression coefficient) AND variation (e.g. standard deviation) or associated estimates of uncertainty (e.g. confidence intervals)
☐	☒	For null hypothesis testing, the test statistic (e.g. *F*, *t*, *r*) with confidence intervals, effect sizes, degrees of freedom and *P* value noted<br>*Give P values as exact values whenever suitable.*
☒	☐	For Bayesian analysis, information on the choice of priors and Markov chain Monte Carlo settings
☒	☐	For hierarchical and complex designs, identification of the appropriate level for tests and full reporting of outcomes
☐	☒	Estimates of effect sizes (e.g. Cohen's *d*, Pearson's *r*), indicating how they were calculated

Our web collection on statistics for biologists contains articles on many of the points above.

Software and code

Policy information about availability of computer code

Data collection	Microsoft Excel & Word-Microsoft Office 2017; Image J 1.52g, NIH; EndNote X9, Thomson Router; R (V 2.15.2); Olympus Confocal FV3000, Olympus; Olympus fluorescence microscope IX70, Olympus; Olympus optical microscope CX23, Olympus; ACCU-CHEK Performa, Roche Diabetes Care GmbH; ABI PRISM 7900HT Detection Systems, Applied Biosystems
Data analysis	SPSS (Statistical Product and Service Solutions) Statistics Software (Version 25.0, Mac OS X Snow Leopard; IBM);<br>Image (Version J 1.52g, NIH) was used for quantification of positive-staining cells in the immunofluorescence staining and cell diameter of adipocytes;<br>ABI PRISM 7900HT detection systems was used for collection of qPCR data;<br>GraphPad Prism Software (Version 9.4.1.681 for Microsoft Windows; GraphPad Software, San Diego, USA) was used for data analysis.

For manuscripts utilizing custom algorithms or software that are central to the research but not yet described in published literature, software must be made available to editors and reviewers. We strongly encourage code deposition in a community repository (e.g. GitHub). See the Nature Portfolio guidelines for submitting code & software for further information.

Data

Policy information about availability of data

All manuscripts must include a data availability statement. This statement should provide the following information, where applicable:

- Accession codes, unique identifiers, or web links for publicly available datasets
- A description of any restrictions on data availability
- For clinical datasets or third party data, please ensure that the statement adheres to our policy

The clinical and histological characteristics of the human samples are provided with this paper.

All other data generated or analyzed during this study are included in this published article and its supplementary information files.
Source data are provided with this paper.

Field-specific reporting

Please select the one below that is the best fit for your research. If you are not sure, read the appropriate sections before making your selection.

☒ Life sciences ☐ Behavioural & social sciences ☐ Ecological, evolutionary & environmental sciences

For a reference copy of the document with all sections, see nature.com/documents/nr-reporting-summary-flat.pdf

Life sciences study design

All studies must disclose on these points even when the disclosure is negative.

Sample size	Sample size was chosen taking in consideration the means of the target values between the experimental group and the control group, the mean standard error and the statistical analysis used. For animal studies, sample size was defined on the basis of past experience with the models, to allow a power ≥80% at the 5% significance level. For ethical reasons, the minimum number of animals necessary to achieve the scientific objectives was used. The determination and justification of animal numbers for this work is performed according to the Laboratory Animal -Guide Line for Ethical Review of Animal of China (GB/T 35892-2018) and the Academic Committee of Experimental Animal Ethics, Use & Care Union in Chongqing University of Education.
Data exclusions	No data were excluded in the current work when performing the final statistical analysis,which have been provided state in "Statistical analysis" section.
Replication	All in vitro experiments were performed in triplicate unless specified in the figure legends. The detailed replication of each experiments has been provided in Figure Legend.
Randomization	For animal experiments, age-matched mice with different genotypes were randomly divided into different experimental groups. For cell culture experiments, cells with different genotypes or treatments were randomly divided into different experimental groups.
Blinding	The investigators were unaware of the experimental groups in all the quantifications.

Reporting for specific materials, systems and methods

We require information from authors about some types of materials, experimental systems and methods used in many studies. Here, indicate whether each material, system or method listed is relevant to your study. If you are not sure if a list item applies to your research, read the appropriate section before selecting a response.

Materials & experimental systems

n/a	Involved in the study	
☐	☒	Antibodies
☐	☒	Eukaryotic cell lines
☒	☐	Palaeontology and archaeology
☐	☒	Animals and other organisms
☐	☒	Human research participants
☒	☐	Clinical data
☒	☐	Dual use research of concern

Methods

n/a	Involved in the study	
☒	☐	ChIP-seq
☒	☐	Flow cytometry
☒	☐	MRI-based neuroimaging

Antibodies

Antibodies used	Corresponding primary antibodies used in the study and against the following indicated proteins were purchased from Abcam Inc: anti-Actin (#ab179467, 1/2500 dilution), anti-GAPDH (#ab9485, 1/1500 dilution), anti-CEBP delta (#ab245214, 1/1000 dilution), anti-HIF-1 alpha (#ab179483, 1/1000 dilution), anti-iNOS (#ab178945, 1/1000 dilution), anti-p38 (#ab182453, 1/1000 dilution), anti-phosphorylated p38 (#ab4822, 1/1000 dilution), anti-NF-κB p65 (#ab32536, 1/1000 dilution), anti-phosphorylated NF-κB p65 (#ab76302, 1/1000 dilution), anti-alpha skeletal muscle actin (#ab184705, 1/1000 dilution), anti-CD11b (#ab133357, 1/100 dilution) and anti-F4/80 (#ab16911, 1/100 dilution). Antibodies against TRIM26 (#PA5-62191, 1/150-1/1000 dilution) and TRIM26 (#ABIN7076014, 1/150-1/1000 dilution) were obtained from Thermo Fisher Scientific and Antibodies-online GmbH Inc., respectively. Moreover, the antibodies against anti-Flag (CST, #14793, 1/1000 dilution), anti-HA (Abcam, #ab9110, 1/1000 dilution), anti-Ub (Abcam, #ab134953, 1/1000 dilution) and anti-Myc (Abcam, #ab9106, 1/1000 dilution) were also used in the current study. The QuantiPro™ BCA protein quantification kits (#71285-3, Millipore®) was used to determine samples' protein concentration. The HRP-tagged secondary antibodies (Thermo Fisher Scientific) with 1/10,000-1/15,000 dilution was used for visualization in western blotting analysis. TaqMan® Universal PCR Master Mix (#P/N 4304437) and PowerUp™ SYBR™ Green (#A25742) were purchased from Applied Biosystems.
Validation	All antibodies used in our study have been validated and detailed information could be found on the websites from manufactures as

listed below:
anti-Actin (#ab179467, 1/2500 dilution): https://www.abcam.cn/products/primary-antibodies/actin-antibody-epr16769-ab179467.html
anti-GAPDH (#ab9485, 1/1500 dilution): https://www.abcam.cn/products/primary-antibodies/gapdh-antibody-loading-control-ab9485.html
anti-CEBP delta (#ab245214, 1/1000 dilution): https://www.abcam.cn/products/primary-antibodies/cebp-deltacebpd-antibody-epr23518-259-ab245214.html
anti-HIF-1 alpha (#ab179483, 1/1000 dilution): https://www.abcam.cn/products/primary-antibodies/hif-1-alpha-antibody-epr16897-ab179483.html
anti-iNOS (#ab178945, 1/1000 dilution): https://www.abcam.cn/products?keywords=%23ab178945
anti-p38 (#ab182453, 1/1000 dilution): https://www.abcam.cn/products/primary-antibodies/p38-alphamapk14-antibody-epr16878-ab182453.html
anti-phosphorylated p38 (#ab4822, 1/1000 dilution): https://www.abcam.cn/products/primary-antibodies/p38-phospho-t180--y182-antibody-ab4822.html
anti-NF-κB p65 (#ab32536, 1/1000 dilution): https://www.abcam.cn/products/primary-antibodies/nf-kb-p65-antibody-e379-ab32536.html
anti-phosphorylated NF-κB p65 (#ab76302, 1/1000 dilution): https://www.abcam.cn/products/primary-antibodies/nf-kb-p65-phospho-s536-antibody-ep2294y-ab76302.html
anti-alpha skeletal muscle actin (#ab184705, 1/1000 dilution): anti-CD11b (#ab133357, 1/100 dilution): https://www.abcam.cn/products/primary-antibodies/alpha-skeletal-muscle-actin-antibody-epr18430-ab184705.html
anti-F4/80 (#ab16911, 1/100 dilution): https://www.abcam.cn/products/primary-antibodies/f480-antibody-bm8-ab16911.html
anti-TRIM26 (#PA5-62191, 1/150-1/1000 dilution): https://www.thermofisher.cn/cn/zh/antibody/product/TRIM26-Antibody-Polyclonal/PA5-62191
anti-TRIM26 (#ABIN7076014, 1/150-1/1000 dilution): https://www.antibodies-online.com/antibody/7046946/anti-Tripartite+Motif+Containing+26+TRIM26+antibody/
anti-Flag (CST, #14793, 1/1000 dilution): https://www.cellsignal.com/products/primary-antibodies/dykddddk-tag-d6w5b-rabbit-mab-binds-to-same-epitope-as-sigma-s-anti-flag-m2-antibody/14793
anti-HA (Abcam, #ab9110, 1/1000 dilution): https://www.abcam.cn/products/primary-antibodies/ha-tag-antibody-chip-grade-ab9110.html
anti-Ub (Abcam, #ab134953, 1/1000 dilution): https://www.abcam.cn/products/primary-antibodies/ubiquitin-antibody-epr8830-ab134953.html
anti-Myc (Abcam, #ab9106, 1/1000 dilution): https://www.abcam.cn/products/primary-antibodies/myc-tag-antibody-ab9106.html
The QuantiPro™ BCA protein quantification kits (#71285-3, Millipore®):https://www.sigmaaldrich.cn/CN/zh/product/mm/71285m
The HRP-tagged secondary antibodies (Thermo Fisher Scientific) with 1/10,000-1/15,000 dilution:
https://www.thermofisher.cn/cn/zh/antibody/product/Goat-anti-Mouse-IgG2a-Secondary-Antibody-Polyclonal/PA1-28885
https://www.thermofisher.cn/cn/zh/antibody/product/Goat-anti-Human-IgG-F-ab-2-Secondary-Antibody-Polyclonal/31482
https://www.thermofisher.cn/cn/zh/antibody/product/Goat-anti-Rabbit-IgG-F-ab-2-Secondary-Antibody-Polyclonal/31461
TaqMan® Universal PCR Master Mix (#P/N 4304437): https://www.thermofisher.cn/order/catalog/product/4304437?SID=srch-srp-4304437
PowerUp™ SYBR™ Green (#A25742): https://www.thermofisher.cn/order/catalog/product/A25742?SID=srch-srp-A25742

Eukaryotic cell lines

Policy information about cell lines

Cell line source(s)	Human L02 cells were purchased from the Type Culture Collection of the Chinese Academy of Sciences (Shanghai, China), which have been provided in "Cell Culture and Treatment" section.
Authentication	Human L02 cell line were verified by short tandem-repeat DNA profiling before the study.
Mycoplasma contamination	Human L02 cell line were used immediately after being received he Type Culture Collection of the Chinese Academy of Sciences (Shanghai, China) and certified as Mycoplasma free.
Commonly misidentified lines (See ICLAC register)	No misidentified cell lines were used in this study.

Animals and other organisms

Policy information about studies involving animals; ARRIVE guidelines recommended for reporting animal research

Laboratory animals	To minimize the effects of hormones oscillation on metabolism, only male animals (6-8 weeks old) were used for all experiments in this work. Before the experiment starts, animals participating in the corresponding experiment were forced to accommodate to their living environment for 7 days. The animals were kept at a steady temperature, moisture capacity (governed by Haier central air conditioning, Cat: RFC140MXSCVD/G, China), and aseptic conditions-controlled environment (25°C, 55 %-60 %) cage with a constant and standard 12/24 h-12/24 h light/dark circle, unlimited pathogen-free-drinking water (Cat: 1010004000, C'estbon, China) and fodder in their houses. For rabbit in vivo experiment, according to previous report with certain modification (26-28), 20 male New Zealand white rabbits (1.75-2.00 kg BW) were treated with corresponding HFHC diet (standard diet with an additional 2% maltodextrin, 2% cholesterol, and 10% saturated fats, Cat: 621079; Dyets, Bethlehem, Pa) for 8 weeks to establish rabbit NASH model. The rabbits were kept at a steady temperature, moisture capacity (governed by Haier central air conditioning, Cat: RFC140MXSCVD/G, China), and aseptic conditions-controlled environment (25°C, 55 %-60 %) cage with a constant and standard 12/24 h-12/24 h light/dark circle, unlimited pathogen-free-drinking water (Cat: 1010004000, C'estbon, China) and fodder in their houses.

Trim31flox/flox mice based on C57BL/6N background were generated using CRISPR/Cas9-mediated genome engineering system. Exons 4 and 5 of Trim31 were then selected as conditional knockout region (CKO). In brief, the chosen exons of Trim31 were flanked by loxP sites, and therefore two single guide RNAs (gRNA1 and gRNA2) targeting Trim31 introns were designed. The targeting vector containing Trim31 exon 4 and 5 flanked by two loxP sites and the two homology arms were used as the template. The targeting vector, guide RNA1 and guide RNA2 and Cas9 mRNAs were co-injected into fertilized eggs for CKO mouse production. The obtained mice, which had exon 4 and 5 flanked by two loxP sites on one allele, were used to construct Trim31flox/flox mice. Hepatocyte-specific Trim31 deletion (THKO) mice were created by mating Trim31flox/flox mice with albumin-Cre (Alb-Cre) mice (Jackson Laboratory, Bar Harbor, Maine, USA). A simple schematic diagram has been indicated in Supplementary Fig. S2a. Trim31flox/flox mice littermates were used in the study as controls for the obtained THKO mice.

The hepatocyte-specific Rhbdf2-knockout (RHKO) mice were also created using CRISPR/Cas9 system by specifically ablating the 4th exon of Rhbdf2 in hepatocytes. Detailed protocols and information regarding the establishment and genotype determination of these mice have been described previously 34. In addition, the hepatocyte-specific Trim31 and Rhbdf2 double deletion (DHKO) mice were generated by crossing Trim31flox/flox mice with RHKO mice.

Conditional Trim31 transgenic (TG) mice were established by micro-injecting CAG-loxP-CAT-loxP-Trim31 into fertilized eggs isolated from C57BL/6 mice. The obtained pups were then genotyped by PCR followed by sequencing analysis. The obtained mice were identified by PCR analysis of tail genomic DNA. The offspring of these TG mice were mated with the Alb-Cre mice to establish hepatocyte-specific Trim31 transgenic (THTG) mice. The corresponding littermates without Trim31 overexpression in hepatocytes were used as controls (NTG). Additionally, all the other normal wild-type (WT) C57BL/6N mice used in the current study were purchased from Beijing Vital River Laboratory Animal Technology Co., Ltd. (Beijing, China).

Wild animals	No wild animals were used in the study.
Field-collected samples	No field-collected samples were used.
Ethics oversight	All mouse experiments and procedures were reviewed and approved by the Institutional Animal Care and Use Committee (IACUC) at the Chongqing Key Laboratory of Medicinal Resources in the Three Gorges Reservoir Region and other participating units.

Note that full information on the approval of the study protocol must also be provided in the manuscript.

Human research participants

Policy information about studies involving human research participants

Population characteristics	Human liver tissue samples were collected painlessly from adult patients with nonalcoholic fatty liver disease who underwent liver transplantation or liver biopsy. The corresponding control liver tissue was harvested from the donor who could not be used for liver transplantation due to non-hepatic reasons. Written informed consent was integrally obtained from the liver donors and their families. The patient characteristics and liver injury-associated serology were showed in Supplementary table 1.
Recruitment	Human donors' liver specimens were harvested from adult donors with NAFLD who underwent biopsy tissue samples or liver transplantation samples. The relevant non-steatotic liver tissues were obtained from donors who were not eligible for liver transplantation for non-liver reasons. Non-steatosis samples (n=16), Simple steatosis samples (n=17), and NASH phenotype liver samples (n=16) were obtained and included in this study. Of note, steatotic liver samples from patients with any of the following conditions were excluded from the study: excessive drinking (alcohol >70 g for female or alcohol >140 g for male, per week), viral infection or drug abuse (including hepatitis B & C virus infection). Samples from non-steatotic liver were collected from the normal region of the livers from donors who received liver resection owing to liver hemangioma or cyst of liver. Hierarchical steatosis and steatohepatitis were independently diagnosed by two pathologists according to the scoring system of standard histological criteria established by the NASH Clinical Research Network. Cases with NAFLD activity scores (NAS) of 1–2, and ballooning scores of 0 and no fibrosis, were classified as simple steatosis. Cases with NAS ≥ 5 or NAS of 3–4 but with fibrosis were classified as NASH. Cases with NAS of 0 were classified as normal. Also, prior to this study, the samples of non-steatosis and simple steatosis in this cohort were collected from patients without taking statins or insulin. The NASH phenotype liver samples (n=16) in this cohort were from patients who had taken pioglitazone (15-30 mg/day) for no more than 24 months. Liver sample donors and their families agree & sign written informed consent. Physiological characteristics of patients and hepatic injury-related serology are shown in Supplementary information. All protocols involving human donors in this work were grounded on the Ethical Principles for Medical Research Involving Human Subjects, Declaration of Helsink (64th WMA general assembly), and totally approved by the Academic Committee of Experimental Animal Ethics, Use & Care Union in Chongqing University of Education, Chongqing University, Shandong Cancer Hospital and Institute, Shandong First Medical University & Shandong Academy of Medical Science, Shandong University of Traditional Chinese Medicine, Third Military Medical University (Army Medical University) and Fudan University.
Ethics oversight	The whole experimental protocols regarding animals used in this study were permitted by the Guide for the Care & Use of Laboratory Animals (8th edition NIH, in Chinese) and permitted by the Institutional Animal Use & Care Committee (IACUC) in Chongqing University of Education (20190012CQUE). The approaches and procedures involved in current work were used in line with the Regulations of the People's Republic of China on the Administration of Experimental Animals (Revised & Exposure Draft), issued by the Ministry of Science and Technology of the People's Republic of China (http://www.most.gov.cn). All protocols in this work were grounded on the Ethical Principles for Medical Research Involving Human Subjects, Declaration of Helsink (64th WMA general assembly), and totally approved by the Academic Committee of Experimental Animal Ethics, Use & Care Union in Chongqing University of Education, Chongqing University, Shandong Cancer Hospital and Institute, Shandong First Medical University & Shandong Academy of Medical Science, Shandong University of Traditional Chinese Medicine, Third Military Medical University (Army Medical University) and Fudan University.

Note that full information on the approval of the study protocol must also be provided in the manuscript.
